# Advancing Bidirectional Photoswitching of Norbornadienes: Exclusively Light‐Induced Interconversion of *Imide*‐ and *Ortho*‐Connected Norbornadiene‐Perylene Diimide Hybrids

**DOI:** 10.1002/chem.202502610

**Published:** 2025-10-24

**Authors:** Simone Pintér, Nina M. Strassner, Daniel Krappmann, Erik J. Schulze, Andreas Hirsch

**Affiliations:** ^1^ Chair of Organic Chemistry II, Department of Chemistry and Pharmacy Friedrich‐Alexander Universität Erlangen‐Nürnberg Nikolaus‐Fiebiger‐Strasse 10 Erlangen 91058 Germany

**Keywords:** bidirectional photoconversion, energy storage, molecular photoswitches, MOST, norbornadiene

## Abstract

The norbornadiene/quadricyclane (NBD/QC) photoswitch is a promising candidate for molecular solar energy storage. Isomerization to the energy‐storing, metastable QC occurs upon UV light irradiation, while the back‐switching can be triggered in various ways. However, inducing complete, on‐demand energy release across multiple switching cycles remains a significant challenge in molecular solar thermal (MOST) research. We demonstrate the implementation of perylene diimide (PDI) as covalently connected, photoactive redox‐catalyst to enable NBD regeneration upon 475 nm irradiation. We investigated the NBD/QC interconversion of several *imide*‐ and *ortho*‐connected NBD‐PDI hybrids upon irradiation at 310 nm and 475 nm, revealing that the connection position on PDI only minimally affects the interconversion efficiency. Concentration‐dependent studies identified an alternative intermolecular back‐isomerization mechanism in addition to the previously known intramolecular pathway, for which adequate flexibility of the linker proved crucial for effective back‐isomerization to NBD. This study advances the exclusively photoinduced isomerization of the NBD/QC system within novel NBD‐PDI dyads, establishing a framework for the targeted manipulation of NBD hybrids with potential application in energy storage. The presented switches operate independently, without the need for further additives to facilitate interconversion, which renders them promising candidates for molecular solar energy storage research.

## Graphical abstract

Bidirectional Photoswitching: We report norbornadiene‐perylene diimide (NBD‐PDI) dyads with diverse connectivities, enabling exclusively photoinduced NBD/QC switching. Our findings reveal high conversion yields in both directions, paired with good thermal‐ and photo‐stability. Rapid back‐isomerization to NBD occurs upon visible light irradiation, highlighting the potential for on‐demand energy release in energy storage applications.

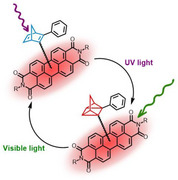

## 1 Introduction

**Figure 1:**
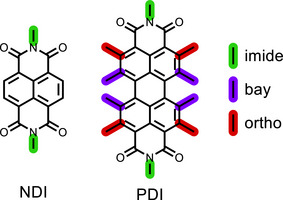
Molecular structures of NDI and PDI. The substitution positions are color coded.

Nowadays, heat production alone accounts for 39% of the global energy‐related CO_2_ emissions, representing a significant share of the annual greenhouse gas output.^[^ ^1^, ^2^ ^]^ The implementation of sustainable heating systems has stagnated in recent years due to high initial investment costs.^[^ ^3^ ^]^ To counteract a return to fossil fuel‐based energy sources, the development of efficient alternative sustainable heating systems is crucial. In recent years, the norbornadiene (NBD) and quadricyclane (QC) interconversion couple received great attention as a prominent representative of molecular solar thermal (MOST) energy storage systems.^[^ ^4^, ^5^, ^6^ ^]^ MOST compounds are designed to harvest solar energy and directly store it in the form of chemical energy, which can subsequently be released on demand in the form of heat. Hereby, the NBD/QC couple is an intriguing candidate due to its high energy storage density of 89 kJ/mol.^[^ ^7^ ^]^ Upon irradiation with UV light, a [2 + 2] cycloaddition is induced, converting NBD to its metastable isomer QC.^[^ ^8^ ^]^ The thermal back‐conversion is forbidden by Woodward‐Hoffmann rules, resulting in excellent thermal stability and extended storage times (half‐lives > 14 hours at 140 °C).^[^ ^9^ ^]^ Although the investigation of the NBD/QC photoisomerization dates back to the 1950s, several obstacles have yet to be overcome to pave the way for practicable application.^[^ ^10^, ^11^ ^]^ Specifically, the design of a trigger that facilitates rapid and complete energy release poses one of the major challenges. Heat‐release can be accomplished, for example, through electrochemical oxidation of QC^[^ ^12^ ^]^ or transition metal catalysis.^[^ ^13^, ^14^ ^]^ However, there are only a few reports of direct photochemical back‐isomerization of QC due to the oftentimes limited optical separation of the NBD and QC absorption and the need for short UV irradiation wavelengths, which is commonly accompanied by photodegradation.^[^ ^5^, ^15^ ^]^ These challenges can be circumvented by the introduction of a photoactive, electron‐accepting unit which initiates the QC‐to‐NBD conversion through charge transfer. This was first demonstrated by our group using C_60_‐NBD hybrids in which the regeneration of NBD was facilitated upon irradiation of the C_60_‐core using wavelengths of lower energy than required for the prior QC formation.^[^ ^16^ ^]^ Subsequent studies reported similar interconversion dyads using naphthalene diimide (NDI) and perylene diimide (PDI) as photoactive redox catalysts (Figure 1).^[^ ^17^, ^18^ ^]^

**Scheme 1:**
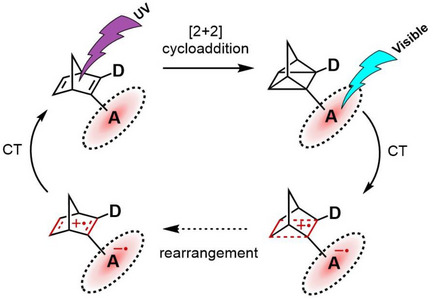
Schematic representation of the solely photoinduced conversion mechanism between NBD and QC using an electron‐accepting photoactive redox catalyst (A). Upon irradiation with UV light, a [2 + 2] cycloaddition converts NBD to QC; 475 nm irradiation induces an electron transfer (CT) from QC to A, facilitating the rearrangement to oxidized NBD followed by a charge back‐transfer. D represents an electron‐donating moiety.

Although the substitution of NBD reduces its exceptional energy storage density, a comprehensive understanding of the photoinitiated isomerization offers valuable insights into the underlying mechanism. In this regard, a systematic investigation of rylene‐NBD dyads holds great potential for establishing a foundation for future targeted manipulation of NBD, particularly in the context of MOST research.^[^ ^8^ ^]^ Previously, we investigated the linker dependency of *imide*‐substituted NDI‐NBD hybrids, observing a pronounced effect of the linker length on the isomerization ratio.^[^ ^18^ ^]^ However, the NBD‐centered absorption was partially superimposed by NDI‐based absorptions, thus resulting in incomplete isomerization and the rapid formation of photostationary states (PSS).^[^ ^18^ ^]^ In a subsequent study, zika et al. advanced the rylene‐NBD strategy by replacing NDI with PDI, which exhibits absorption maxima in the visible region. The reported *bay*‐substituted NBD‐PDIs demonstrated improved spectral separation of NBD‐ and PDI‐centered absorption, enabling back‐isomerization under 475 nm irradiation in contrast to previously required 400 nm and 365 nm LEDs.^[^ ^17^ ^]^ The proposed QC‐to‐NBD mechanism is consistent across all systems: excitation of the photoactive electron acceptor initiates a charge transfer from QC to the extended π‐system, facilitating the rearrangement of oxidized QC to oxidized NBD, which is then reduced (Scheme 1). Previous computational investigations corroborated a facile back‐conversion through a radical cationic QC state.^[^ ^19^ ^]^

Nevertheless, the precise mechanism underlying the interconversion has not yet been unraveled. Previous studies have shown that the PDI substitution pattern significantly affects its charge transfer mobility and its overall electronic structure.^[^ ^20^ ^]^ Hence, the question arises if and how the connectivity of NBD to the PDI core affects the interconversion. In this study, we present the synthesis and photophysical characterization of NBD‐PDI dyads with NBD units linked either at the *imide*‐ or *ortho*‐positions of the PDI core. Synthetic strategies were developed for both PDI positions, employing two distinct types of linkers in each position. All precursors and target compounds were characterized using NMR, UV/Vis, and fluorescence spectroscopy, as well as high‐resolution mass spectrometry (HRMS). Irradiation studies were conducted to evaluate the impact of different linkers and connection positions on the conversion efficiency. To supplement the findings, computational investigations were conducted by triple‐zeta DFT calculations to further study the ground‐state geometries and the influence of the substitution pattern on the frontier molecular orbitals (FMOs).

## 2 Results and Discussion

### 2.1 Synthesis of NBD‐PDI Hybrid Structures

**Scheme 2:**
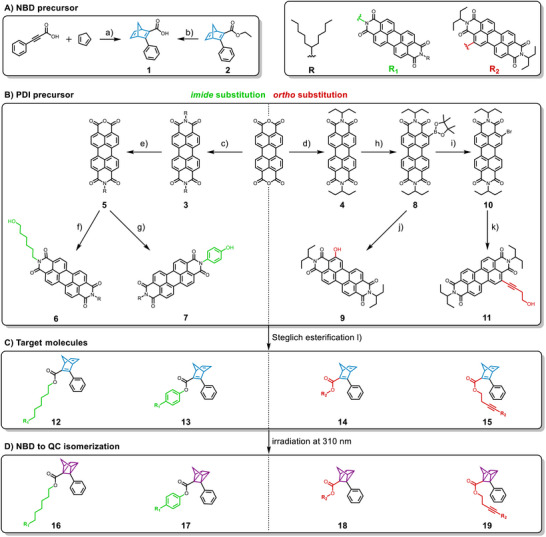
**A)** Synthesis of the NBD precursor **1**: a) phenylpropiolic acid, cyclopentadiene, toluene (Tol), 110 °C, 16 hours, mw; b) **2**, NaOH, THF/H_2_O 1:1 (v/v), 100 °C, 21 hours, mw. **B)** Synthetic scheme toward PDI precursor derivatives **6**, **7**, **9,** and **11** starting from perylenetetracarboxylic dianhydride (PTCDA): c) 6‐aminoundecane, imidazole, 130 °C, 4 hours; d) 3‐aminopentane, imidazole, 100 °C, 3 hours; e) **3**, KOH, *t*‐BuOH, reflux, 2.5 hours; f) **5**, 6‐aminohexanol, imidazole, 180 °C, 5 hours; g) **5**, 4‐aminophenol, imidazole, 180 °C, 4 hours; h) **4**, B_2_pin_2_, Ir[(OMe)cod]_2_, P(C_6_F_5_)_3_, 1,4‐dioxane, 110 °C, 70 hours; i) **8**, CuBr_2_, dioxane/MeOH/H_2_O 5:2:1 (v/v/v), 120 °C, 18 hours; j) **8**, NaOH, NH_3_OHCl, EtOH, rt, 18 hours; k) **10**, 3‐butyn‐1‐ol, Pd(PPh_3_)_4_, CuI, NEt_3_, THF, rt, 5 hours. **C**) Synthesis of the target molecules **12–15**: l) **1**, *N*,*N*′‐dicyclohexylcarbodiimide (DCC), dimethylaminopyridine (DMAP) in dichloromethane (DCM) at reflux for 17–52 hours. **D)** Conversion to their QC isomers upon irradiation at 310 nm.

The target molecules were designed by adapting the synthetic strategy utilized for previously reported rylene‐NBD systems.^[^ ^17^, ^18^ ^]^ The respective characterization can be found in the Supporting Information. The NBD precursor **1** bearing a carboxylic acid and a phenyl moiety was synthesized via two distinct procedures (Scheme 2A). This specific NBD substitution pattern was chosen in accordance with previous work for better comparability. Following a literature procedure, **1** was isolated in 64% yield by the saponification of NBD **2**, which can be produced on a gram scale via a diels‐alder reaction.^[^ ^14^, ^16^ ^]^ Moreover, a second synthetic route was developed, generating **1** directly from phenylpropiolic acid and cyclopentadiene through a diels‐alder reaction using microwave irradiation with a yield of 39%.

The synthesis of the PDI alcohol precursors is depicted in Scheme 2B. Functionalization at the *imide*‐position follows a straightforward procedure, compatible with a broad range of substituents. Compounds **3** and **4** are formed stoichiometrically during the imidation of perylene tetracarboxylic acid (PTCDA) with the respective amines in an imidazole melt.^[^ ^21^ ^]^ The introduction of branched long‐chain alkyl chains enhances the solubility by preventing strong π–π interactions.^[^ ^21^ ^]^ Compound **5** was formed by selective hydrolysis of **3** with concentrated KOH in *t*‐BuOH in 48% yield.^[^ ^22^ ^]^ A second imidation of **5** using the desired amino alcohol gave the flexible, hexyl‐linked PDI precursor alcohol **6** and the rigid, phenyl‐linked analogue **7** in yields of 15% and 74%, respectively. The *ortho*‐functionalization proved to be more challenging due to the low reactivity toward electrophilic aromatic substitution at this position. The most prominent method for direct *ortho*‐functionalization involves an iridium‐catalyzed borylation in dioxane. Mono‐*ortho*‐borylated **8** was isolated with a yield of 56%.^[^ ^23^, ^24^, ^25^ ^]^ The *ortho*‐hydroxy PDI precursor **9** was isolated by hydroxylation of **8** in a concentrated ethanolic solution of NaOH in 83% yield. Bromination of **8** with CuBr_2_ in a dioxane/MeOH/H_2_O mixture afforded the *ortho*‐bromo PDI **10** in 68% yield. Subsequently, **10** was submitted to a sonogashira cross‐coupling with 3‐butyn‐1‐ol to form the precursor **11** in 68% yield.^[^ ^23^, ^26^ ^]^ However, conversion of *ortho*‐borylated PDI was consistently accompanied by concurrent protodeborylation, which prevented higher yields. The four target molecules **12**–**15** depicted in Scheme 2C were received by a steglich esterification of NBD precursor **1** with the respective PDI alcohol precursors **6**, **7**, **9**, and **11**, following literature procedures.^[^ ^16^, ^17^, ^18^ ^]^ The conversion to the respective QC isomers **16**–**19** was accomplished upon irradiation with 310 nm LEDs. Characterization was performed by 1D and 2D ^1^H and ^13^C NMR spectroscopy, UV/Vis and fluorescence spectroscopy, and HRMS (see Supporting Information). The photoconversion will be discussed in detail in the subsequent interconversion studies.

### 2.2 Photophysical Characterization

**Figure 2:**
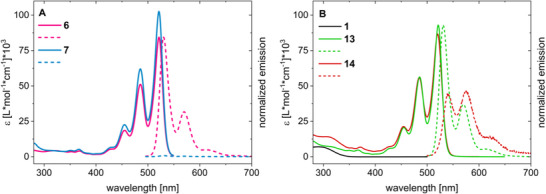
Absorption spectra (solid lines) and emission spectra (normalized; maximum emission of **13** scaled to its absorption; emission of **14** is given relative to **13**) recorded after excitation at 486 nm (dashed lines) of the *imide*‐substituted precursors **6** and **7** (A) and target molecules **13** and **14** (B) in tetrahydrofuran (THF). **1** is given for comparison.

The photophysical properties of the precursor and target compounds were investigated by steady‐state UV/Vis and fluorescence spectroscopy. First, the characteristics of the PDI precursors are discussed based on the example of *imide*‐substituted derivatives **6** and **7**, followed by a comparison with the NBD‐conjugated target compounds **13** and **14**. Both **6** and **7** exhibit the characteristic PDI absorption in the visible region with maxima located at 521 nm, 486 nm, and 455 nm (Figure 2A) in THF. The three peaks originate from 0‐*0, 0‐*1, and 0‐*2 vibrational transitions of the PDI core.^[^ ^17^ ^]^ The absorption between UV and visible region is rather broad and unspecific. Hexanol‐substituted **6** exhibits the anticipated strong fluorescence typical for PDIs, where the emission spectrum appears as a mirror image of the absorption spectrum with a Stokes shift of 9 nm. In contrast, almost no emission is observed for phenol‐substituted **7**. However, after converting **7** to NBD‐linked **13**, the emission spectrum exhibits the expected mirror image of its absorption spectrum once more (Figure 2B). This observation suggests that the phenol substituent is responsible for the absence of fluorescence in **7**. In this context, flamigni et al. reported fluorescence quenching for PDIs substituted with the *para*‐methoxyphenyl analogue of **7**.^[^ ^27^ ^]^ The phenomenon was reasoned by the formation of a charge‐separated state in competition with fluorescent relaxation. In our case, this rationale was substantiated by conducting a solvent‐dependent fluorescence study of **7**. A clear trend of decreasing emission intensity was observed with increasing solvent dipole moment, corroborating the formation of a charge‐separated state as an alternative pathway to a fluorescent decay. The spectra corresponding to the solvent‐dependent study and further UV/Vis and emission spectra of **9** and **11** can be found in the Supporting Information.

Figure 2B depicts the absorption and normalized emission spectra of *imide*‐linked **13** and *ortho*‐linked **14** in comparison to the precursor NBD **1**. Pristine **1** exhibits an absorption maximum at 290 nm and an absorption onset at 353 nm. Dyad **13** features absorption maxima at 522 nm, 486 nm, and 455 nm, and dyad **14** at 520 nm, 485 nm, and 455 nm, which correspond to PDI absorptions. The absorption between 430 and 350 nm is rather broad and unspecific in both compounds. Below 350 nm, **13** and **14** reveal an absorption increase, however, only **14** exhibits a clear maximum at 307 nm. Both emission spectra show the characteristic mirror image shape of the absorption, with Stokes shifts of 9 nm for **13** and 22 nm for **14**. Compared to their precursors **7** and **9** (Supporting Information), the extinction coefficient is lowered by 10%. The absorption increase below 350 nm is observed only in the NBD‐linked target molecules and thus suggested to be mainly NBD‐centered. However, an absorption onset of the exclusively NBD‐related absorption cannot be determined due to superimposition with PDI‐based absorption in the UV‐region. The four hybrids **12**‐**15** exhibit generally similar absorption features and intensities; however, it should be mentioned that the extinction coefficient of **15** is lowered compared to the other dyads (see Supporting Information Figure 31). A similar trend is observed for its precursor **11** compared to the other PDI‐alcohols **6**–**9**. This can be rationalized by a stronger influence of the substituent on the PDI's electronic structure due to the π‐conjugation with the acetylene linker.

**Figure 3:**
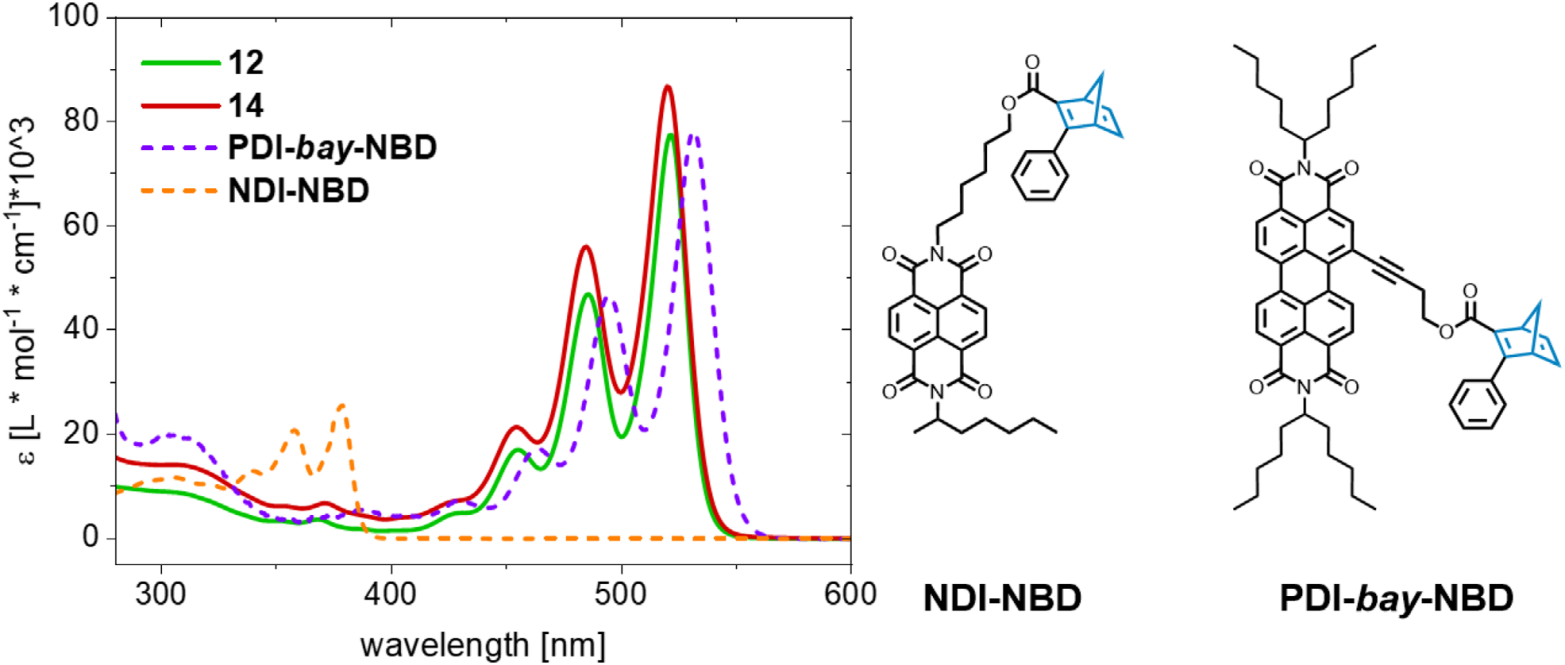
Comparison of the absorption spectra of *imide*‐, and *ortho*‐connected dyads **12** and **14** with previously reported PDI‐*bay*‐NBD (zika et al.)^[^ ^17^ ^]^ and *imide*‐substituted NDI‐NBD (leng et al.)^[^ ^18^ ^].^

A comparison of the extinction coefficients of **12** and **14** with previously reported PDI‐*bay*‐NBD^[^ ^17^ ^]^ and NDI‐NBD^[^ ^18^ ^]^ is depicted in Figure 3. Every NBD‐PDI dyad (**12**, **14**, and PDI‐*bay*‐NBD) reveals a pronounced separation between the PDI‐ and NBD‐centered absorption in contrast to NDI‐NBD. Thus, the specific irradiation of only one component of NBD‐PDIs is feasible regardless of the connection position, substantiating a general advantage of PDI over NDI considering bidirectional convertibility.

However, the intensity of the predominantly NBD‐related absorption at 307 nm in Figure 3 exhibits a notable dependence on the connection position of NBD at the PDI core. Considering that PDI is an excellent electron acceptor, the push‐pull system formed from the ester and phenyl moieties on the NBD double bond might be enhanced by substitution with PDI compared to the free acid **1**. However, no precise statement on the correlation between NBD‐PDI connectivity and NBD absorption features can be made at this point due to the superimposition of PDI and NBD absorption.

### 2.3 NBD/QC Interconversion Monitored by UV/Vis and NMR Spectroscopy

We then conducted irradiation studies to investigate compounds **12**–**15** toward their reversible switching properties. Firstly, the isomerization progress was monitored by UV/Vis spectroscopy. All samples were analyzed in 0.01‐0.08 mM solutions in tetrahydrofuran (THF) and toluene (Tol). Irradiation was performed in a custom‐built irradiation setup with 310 nm and 475 nm LEDs.

**Figure 4:**
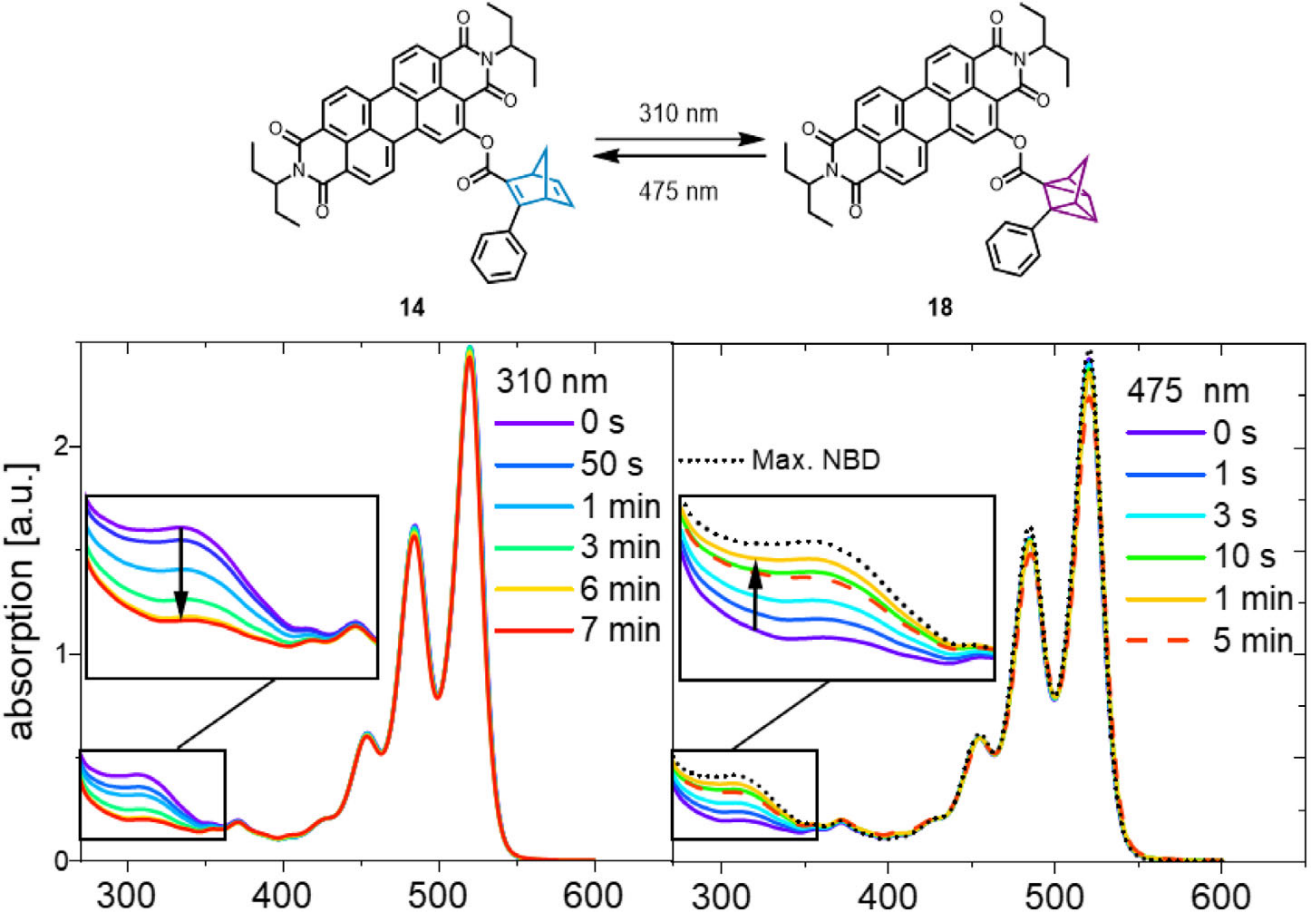
Interconversion study of **14** during irradiation at 310 nm and 475 nm, monitored by UV/Vis spectroscopy. The measurements were conducted in THF (0.05 mM).

Compounds **12**–**15** were successfully converted into their QC‐isomers **16**–**19** (Scheme 2) upon photoirradiation at 310 nm, as evidenced by a decrease of the NBD‐centered absorption. Subsequent irradiation at 475 nm introduces the reverse effect, achieving the desired bidirectional switching of the dyads. An exemplary irradiation study of **14** in THF is displayed in Figure 4. Maximum QC formation was observed after 7 minutes of irradiation at 310 nm, while regeneration of a maximum of only 81% NBD was achieved within 1 minute of irradiation at 475 nm. Extended irradiation at this wavelength led to a reduction of the PDI absorption features, indicating photodecomposition (dashed lines in Figure 4). Due to the PDI and NBD absorption overlapping in the UV region, complete quantitative conversion cannot be proven but was presumed. To enable comparison between the compounds, semiquantitative isomerization ratios were determined based on the absorption difference at 300 nm before and after irradiation at 310 nm. Consequently, the overall change between the first and last measured absorption curve was considered to represent 100% conversion (for details, see Supporting Information). The QC absorption is expected to be located below 250 nm (see irradiation studies of **1** in the Supporting Information) and thus not visible due to the self‐absorption of the utilized solvent. Hence, maximum QC formation is supposed when the spectrum remains unchanged upon prolonged irradiation, and a clean conversion is indicated by consistency in the overall absorption properties.

**Figure 5:**
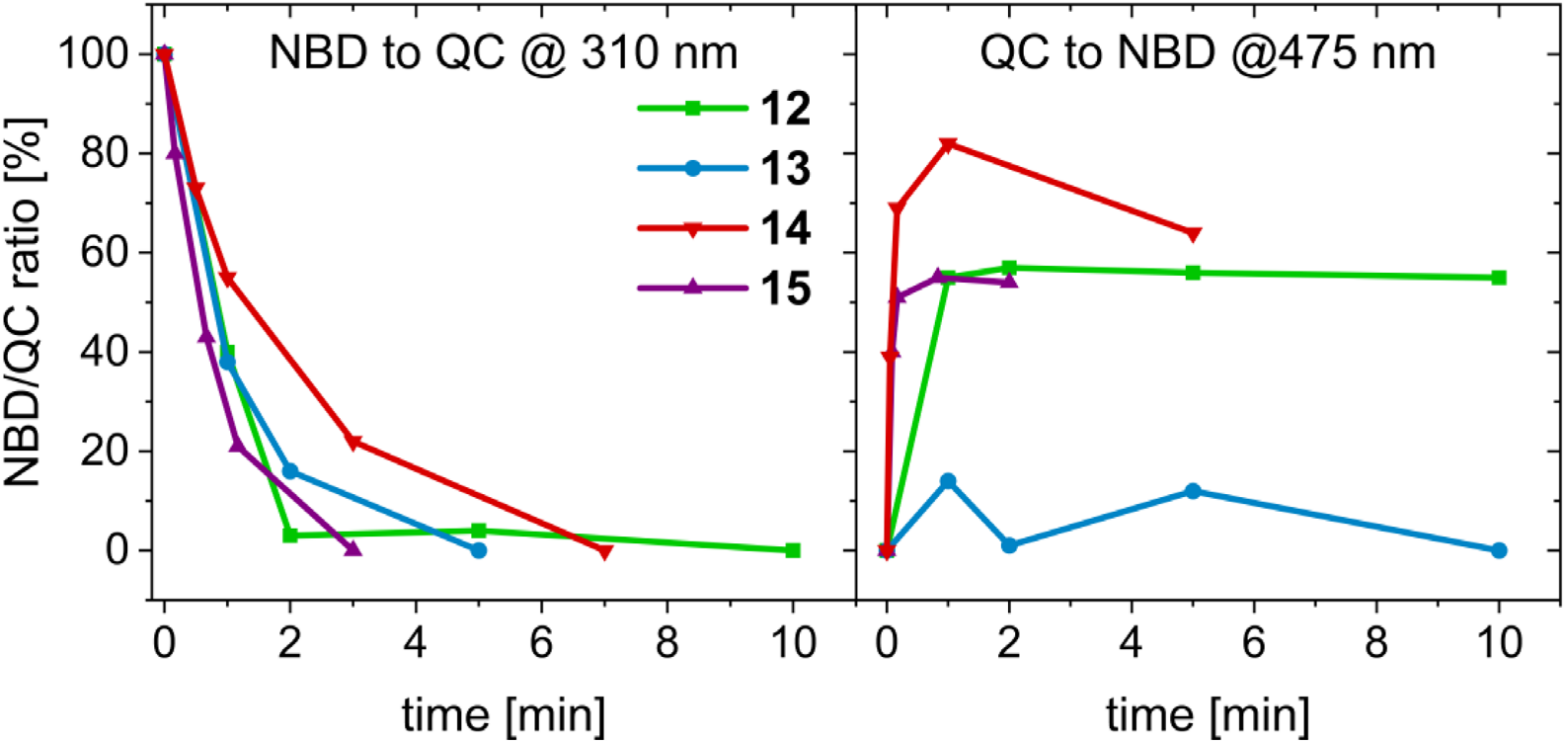
Progression over time of the photoisomerization upon irradiation at 310 nm (left) and 475 nm (right) in THF (0.01–0.05 mM) monitored by UV/Vis.

For all NBD derivatives **12**‐**15**, maximum QC formation upon irradiation was achieved within a few minutes, while the back‐isomerization process occurred at a significantly faster rate, within seconds, using THF as a solvent (Figure 5). However, irradiation at 475 nm resulted in incomplete back‐conversion, establishing a PSS. Notably, compounds **12** and **15**, featuring longer and more flexible hexyl and acetylene linkers, reach their maximum QC isomerization more rapidly than the rigidly connected **13** and **14** in THF. This can either indicate that flexible linkers and thus a less strained system facilitate QC formation or that close spatial proximity of the NBD scaffold and the PDI core promotes QC formation, as it was previously reported to be crucial for NBD regeneration.^[^ ^18^ ^]^ Generally, studies conducted in toluene revealed mostly full NBD regeneration, while this process is hampered in THF (Table 1a). Notably, for **17** almost no back‐conversion was observed in THF. Consequently, further solvent‐dependent irradiation assays of **17** were performed (Table 1b). In toluene and dichloromethane (DCM), full regeneration of **13** is attainable. However, in ethyl acetate (EtOAc) only 74% back‐isomerization is observed, and in THF, the recovery is negligible.

**Table 1:** a) NBD recovery upon irradiation at 475 nm, determined semi‐quantitatively for all four NBD‐PDI dyads **12**–**15**. b) Recovery of **13** in a solvent‐dependence study upon irradiation of **17** at 475 nm.

a] QC to NBD					b] 17 to 13			
	12	13	14	15	Tol	DCM	EtOAc	THF
THF	54%	/	82%	56%	100%	100%	74%	/
Tol	100%	95%	100%	100%				

Such pronounced solvent effects on the interconvertibility of NBD and QC are consistent with previous reports postulating the stabilization of quadricyclane by polar solvents.^[^ ^28^ ^]^ In our case, the hindered recovery of NBD may be attributed to the stabilization of the QC^+^ **^·^** radical by varying degrees of electron‐donation from different solvents. The gutmann's donor number (DN) provides a quantitative measure for comparing the electron pair donation ability of a solvent. Hereby, a higher DN value indicates enhanced donor characteristics. THF has a DN of 20.0, which is 20 and 200 times greater than those of DCM and Tol, respectively. EtOAc also displays strong donor‐characteristics with DN = 17.1, however less pronounced than THF. The observed trend of the DN values aligns well with our semi‐quantitative back‐conversion ratios (Table 1b), offering a plausible, albeit provisional, explanation for our observations.^[^ ^29^ ^]^

**Figure 6:**
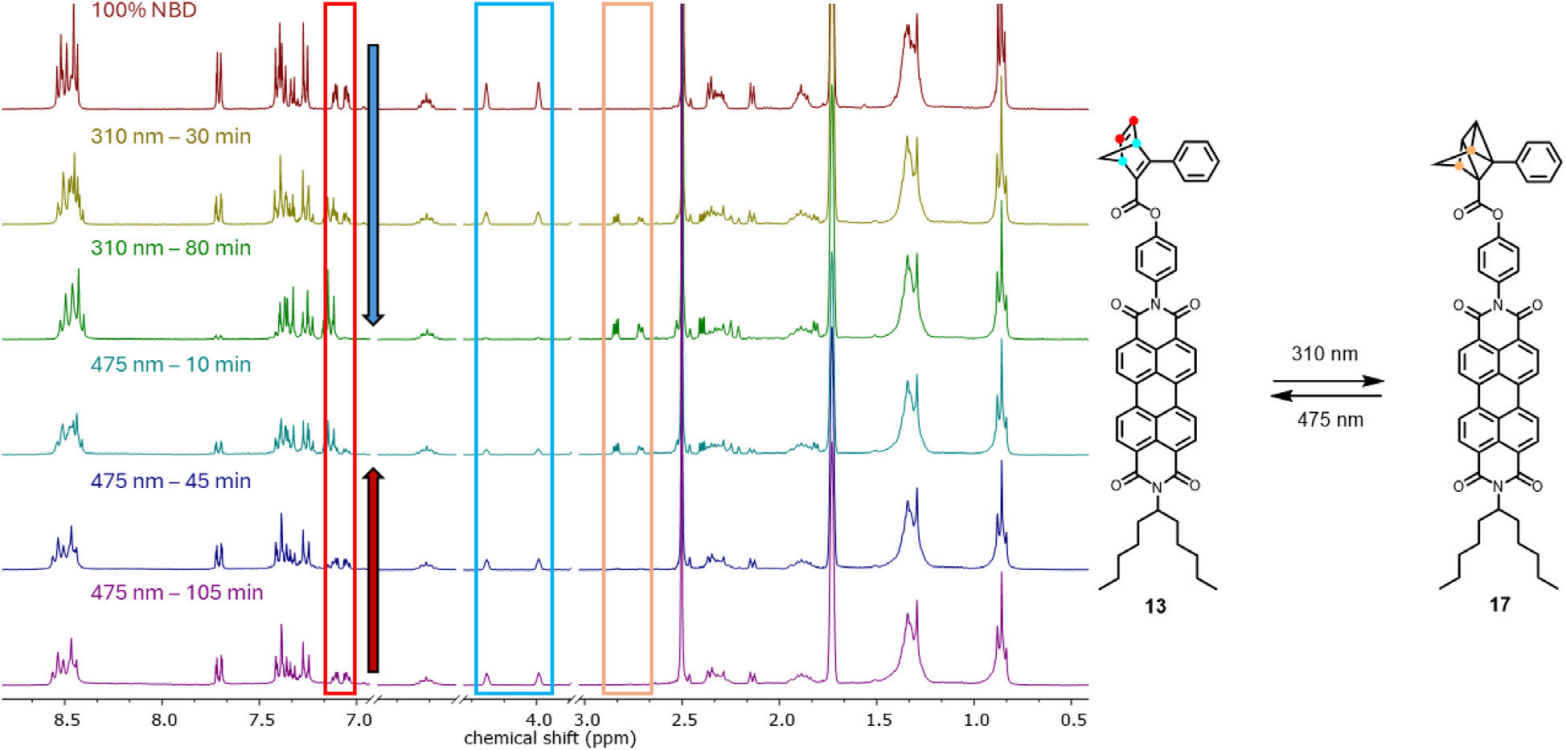
Significant sections of the NMR spectra depicting the isomerization of **13** to **17** at 310 nm and the recovery of **13** at 475 nm irradiation in THF‐*d_8_*. Red = olefinic NBD, blue = bridgehead NBD, and orange = QC signals. The full spectra can be found in the Supporting Information.

Complementary to the UV/Vis assays, irradiation studies were performed, monitored by ^1^H NMR spectroscopy of 5‐8 mM solutions in THF‐*d_8_* and Tol‐*d_8_*. Compared to the UV/Vis studies, longer irradiation times were required for maximum conversions, which is attributed to significantly higher concentrations compared to UV/Vis measurements (5‐8 mM instead of 0.01–0.08 mM) and the different shape of the vessel the irradiation was performed in (5 mm diameter NMR tube instead of 1 cm cuvette). Figure 6 depicts the switching process between **13** and **17** in THF‐*d_8_* followed by ^1^H NMR spectroscopy. QC formation is indicated by the decrease of the olefinic NBD proton signals in the region of 7.12‐7.04 ppm (red box) and the NBD bridgehead proton signals in the region between 4.26‐3.99 nm (blue box) and the simultaneous formation of new QC signals in the region between 2.86‐2.69 ppm (orange box). The reverse observation is made when the compound is irradiated at 475 nm. The olefinic and bridgehead NBD signals are located at similar chemical shifts for every target compound and were applied as spectroscopic markers since they are mostly not superimposed, allowing quantitative monitoring of the isomerization progress.

Irradiation of **13** at 310 nm reveals a maximum conversion of 87% to **17**, establishing a PSS after 80 minutes (Table 2). A rather high NBD regeneration of 92% was observed upon irradiation at 475 nm. No unassignable signals appear, and the remaining spectrum and baseline remain in principle unchanged throughout photoirradiation, giving no indication for decomposition or potential side‐reactions. In the corresponding NMR study conducted in Tol‐*d_8_*, lower QC isomerization yields were observed (79%), while the NBD regeneration was slightly higher than in THF‐*d_8_* (96%), which aligns with the trends found in the UV/Vis studies. Similar NMR assays were conducted for the other derivatives, **12**, **14**, and **15**, yielding comparable results. The corresponding NMR spectra are available in the Supporting Information.

**Table 2:** Maximum isomerization yields upon irradiation with 310 nm (NBD to QC) and 475 nm (QC to NBD) in THF‐*d_8_* (left) and Tol‐*d_8_* (right).

THF‐*d_8_*					Tol‐*d_8_*				
	**12**	**13**	**14**	**15**		**12**	**13**	**14**	**15**
*NBD to QC*					*NBD to QC*				
t [minutes]	120	80	210	120	t [minutes]	120	165	150	120
QC [%]	81	87	85	99	QC [%]	65	79	60	59
*QC to NBD*					*QC to NBD*				
t [minutes]	30	105	29	45	t [minutes]	20	30	20	20
NBD [%]	77	92	67	100	NBD [%]	96	96	100	94

Notably, the observations from NMR and UV/Vis studies regarding the regeneration of compound **13** in THF are markedly different. While no back‐isomerization was detected for this solvent in the UV/Vis assays, almost complete regeneration is found in the NMR study. This discrepancy is attributed to the concentration difference of two orders of magnitude in the two spectroscopic methods. The increased compound concentration in NMR measurements amplifies the probability of intermolecular interactions. It is therefore suggested, that additionally to the so far postulated back‐switching mechanism requiring intramolecular charge transfer, an intermolecularly mediated back‐isomerization is possible. As a reference experiment, isomerization studies of unlinked NBD **1** and precursor PDI **6** were performed and monitored by both UV/Vis and NMR spectroscopy (Supporting Information). Upon irradiation at 310 nm, complete isomerization of **1** was observed using both monitoring methods. The isomerization of **1** monitored by NMR was found to be faster compared to the dyads **12** and **13**. Furthermore, regeneration of **1** upon irradiation at 475 nm was achieved in both low‐concentrated UV/Vis and higher‐concentrated NMR studies, however slower than for the covalently linked hybrids. These findings substantiate the possibility of an intermolecularly catalyzed back‐isomerization process. This realization poses a critical new insight into the so far only partially understood interconversion mechanism of rylene‐NBD hybrids.

**Figure 7:**
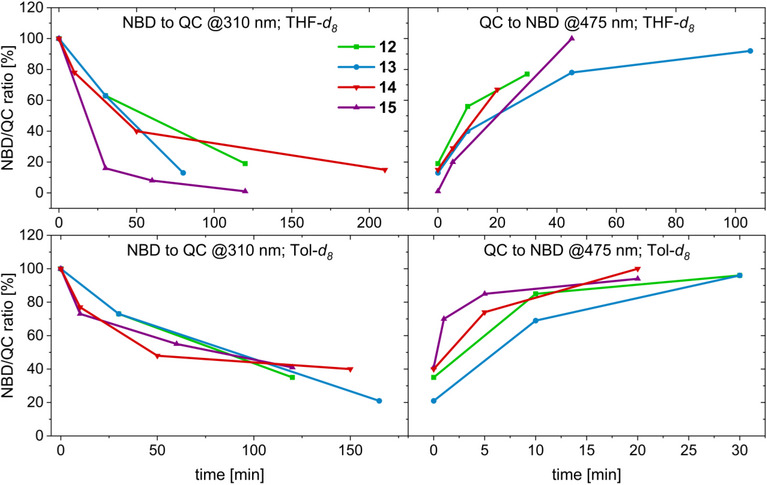
Progression over time of the NMR‐monitored irradiation assays in THF‐*d_8_* (top) and Tol‐*d_8_* (bottom).

The isomerization progress upon irradiation of all synthesized NBD derivatives **12‐15** monitored by NMR is depicted in Figure 7. Notably, all compounds exhibit a similar switching behavior, regardless of the connectivity and linker. In accordance with the UV/Vis studies, solutions in THF‐*d_8_* revealed generally higher QC formation yields but lower back‐conversion ratios than in Tol‐*d_8_*. Almost complete isomerization to **15 **(99%) was achieved in THF‐*d_8_* whereas in Tol‐*d_8_* the best result was observed for **13**, yielding 79% QC isomer. However, high NBD regeneration of more than 94% was possible for all compounds in Tol‐*d_8_*, including quantitative recovery of **14**. In THF‐*d_8_*, back‐isomerization is significantly hampered for **12** and **14,** whereas **13** can be recovered to 92%. Even though the complete vanishing of the QC signals of **15** is observed after 45 minutes of irradiation, the initial NBD signal integrals were not recovered, thus indicating minor decomposition. Detailed information on the studies can be found in the Supporting Information.

Returning to the initial comparison between rylene‐NBD derivatives, PDI proved to be the more efficient photoactive catalyst in the NMR assays. Due to the distinctly separated absorption maxima of NBD and PDI, significantly higher conversion ratios are observed than for the NDI‐predecessors. Notably, **15** reveals a conversion ratio approaching unity, outperforming all other so far reported dyads concerning NBD recovery. However, **13** reveals interesting switching characteristics regarding its consistently high bidirectional interconversion properties in both solvents at higher concentrations, albeit performing no back‐conversion at low concentrations in THF. These findings underline the importance of comprehensive investigations of a diverse range of rylene‐NBDs, as they provide critical insights into the influence of structural elements on the interconversion efficiencies.

### 2.4 Computational Investigations

**Figure 8:**
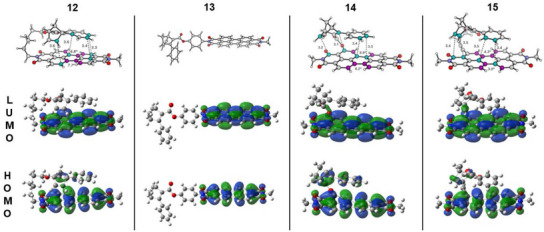
DFT‐optimized geometries of the four synthesized NBD‐PDI dyads with the respective FMOs.

For a deeper understanding of the influence of both the substitution position and the nature of the linker, ground‐state structures were calculated by triple‐zeta DFT calculations including dispersion corrections (B3LYP/6‐311++G(d,p) D3BJ).^[^ ^30^, ^31^, ^32^, ^33^, ^34^, ^35^, ^36^, ^37^ ^]^ CREST conformational searches followed by reoptimization of the lowest energy conformers yielded the structures depicted in Figure 8, representing the NBD ground states.

Given that the input structure from which CREST runs are started can influence the results, two separate searches were performed for **12**: one starting from an “open‐chain” structure that lacked spatial convergence between the NBD and PDI core and another pre‐oriented one. All calculated conformers exhibited a certain degree of π‐interactions between the NBD and PDI, indicating a very strong stabilizing effect originating from these intramolecular interactions. The calculated open‐chain structure demonstrated a free energy difference of + 15.9 kcal/mol compared to the lowest energy conformer obtained in the CREST searches. Thus, it is highly unlikely that the open‐chain structure plays any role in the in situ behavior of this compound. The corresponding QC isomers were calculated based on the previously determined lowest energy NBD conformers. Apart from the phenyl‐linked hybrids, in which intramolecular π‐π interactions are prevented by the rigid linker, all structures exhibited π–π distances ranging from 3.3 to 3.6 Å, which aligns with the distances as, for example, found for two sheets of graphene.^[^ ^38^ ^]^ These findings align with the observations from the irradiation studies, reinforcing the idea that the rigid phenyl linker prevents any intramolecular spatial proximity between the NBD scaffold and the PDI core. Consequently, it is substantiated that intermolecular interactions are essential to facilitate the back‐isomerization regarding compound **17**.

Figure 8 depicts representatively the NBD ground state FMOs of compounds **12**‐**15**. The respective optimized structures and FMOs of **16**–**19** as well as of *bay*‐substituted hybrids and NDI‐NBD can be found in the Supporting Information. The LUMOs of all studied compounds are predominantly localized on the PDI core, with some contribution from the linkers occurring only in the case of *bay*‐ and *ortho*‐substituted compounds. This observation is consistent with previous theoretical studies that report orbital nodes located at the axis between the nitrogens of PDI and the there located moieties, resulting in an electronic decoupling of the *imide*‐substituents.^[^ ^20^ ^]^ However, the LUMOs exhibit no distribution of electron density on NBD and QC in any case, while the HOMOs display involvement of NBD and QC, except for **13**, where the FMOs are entirely localized on the PDI core. Concluding from the similar electron density distributions of the HOMOs of **12**, **14**, and **15**, coupled with the opposite observation in **13**, the involvement of NBD and QC in the HOMOs is attributed to the strong π–π interactions between the NBD‐phenyl and PDI core.

## 3 Conclusion

In this work, we successfully synthesized *imide*‐ and *ortho*‐connected NBD‐PDI derivatives and conducted a comprehensive characterization of their photophysical properties. The spectroscopic investigation was complemented by DFT calculations, elucidating the influence of linker and substitution position on the ground‐state geometries and FMOs of both NBD and QC isomers. Our detailed irradiation studies, monitored by UV/Vis and NMR spectroscopy, shed light onto the influence of the connectivity between PDI and NBD on the photophysical properties and isomerization characteristics of the hybrid systems. The NBD‐PDI dyads reveal generally increased NBD absorption compared to pristine **1**. An intriguing perspective is the introduction of a linker allowing complete conjugation between the PDI's π‐system and the NBD donor‐acceptor system. In the irradiation studies, a sufficiently long and flexible linker was found to be crucial for the back‐isomerization at low concentrations. However, we revealed the existence of intermolecularly mediated back‐isomerization, showcasing an additional pathway to the previously postulated one, which proposed that intramolecular interactions are essential for photocatalytic back‐conversion.^[^ ^18^ ^]^ By expanding the scope of PDI functionalization with NBD, we laid the foundation for a library of rylene‐NBD hybrids and paved the way for further investigations regarding the factors influencing the photophysical properties of such hybrid compounds.

## Supporting Information

The authors have cited additional references within the Supporting Information.^[^ ^39^, ^40^, ^41^, ^42^, ^43^, ^44^, ^45^, ^46^, ^47^, ^48^, ^49^ ^]^

## Conflict of Interest

The authors declare no conflict of interest.

## Supporting information



Supporting Information

## Data Availability

The data that support the findings of this study are available from the corresponding author upon reasonable request.
