# Supplementary material for: Advancing Bidirectional Photoswitching of Norbornadienes: Exclusively Light‐Induced Interconversion of Imide‐ and Ortho‐Connected Norbornadiene‐Perylene Diimide Hybrids
Source: Chemistry. 2025 Oct 24;31(66):e02610. doi: 10.1002/chem.202502610 (PMC12648458; doi:10.1002/chem.202502610)
Supplement: Supplementary file 1 — Supporting Information [file CHEM-31-e02610-s001.docx]

SUPPORTING INFORMATION

[General information 2](#_Toc196853571)

[Syntheses 4](#_Toc196853572)

[NMR spectra 10](#_Toc196853573)

[Fluorescence quenching study 24](#_Toc196853574)

[Absorption and emission data 25](#_Toc196853575)

[UV/Vis assays 26](#_Toc196853576)

[Reference assays of unlinked NBD 1 and PDI precursor 6 31](#_Toc196853577)

[NMR assays 32](#_Toc196853578)

[Computational details 37](#_Toc196853579)

General information

All used chemicals were obtained from common commercial chemical suppliers (Sigma Aldrich, Fisher Scientific, ABCR) unless stated otherwise. Solvents of technical grade were purified by distillation. All NMR spectra were recorded using deuterated solvents which were bought. All reactions were conducted under an inert atmosphere.

TLC analysis was performed on aluminum plates coated with 0.20 mm Merck silica gel 60 containing a fluorescent indicator (Machery-Nagel, ALUGRAM®, SILG/UV254) and visualized by exposure to ultraviolet light (λ = 254 nm and 366 nm). Column chromatography was performed using deactivated silica gel 60M from Macherey-Nagel®, Düren, Germany.

Mass spectrometry (MALDI-TOF or APPI-TOF) was performed on a BRUKER DALTONICS MaXis4g.

UV/Vis spectroscopy was carried out on a Varian Cary 5000 UV/Vis-NIR spectrometer using a high precision cell from Hellma®Analytics (d=1 cm). Fluorescence spectroscopy was performed using an Edinburgh FS5 Emission spectrometer in case of the *ortho*‑ and a Shimadzu RF-5301PC spectroﬂuorophotometer in case of the *imide*‑substituted NBD‑PDIs. Irradiation for UV/Vis measurements was performed in a custom-built irradiation chamber in which the cuvette containing the sample solution was placed (see Figure SI 1). LEDs from Neumüller Elektronik were used in the setup, some inconsistencies were however observed regarding their power within the following ranges:

310 nm: U_applied_ = 4.9-5.3 V; I_measured_ = 0.48-0.56 A; P_max_ = 30 mW @ I_max_ = 0.60 A

475 nm: U_applied_ = 2.9-3.2 V; I_measured_ = 0.27-0.36 A; P_max_ = 3.0 W @ I_max_ = 0.35 A


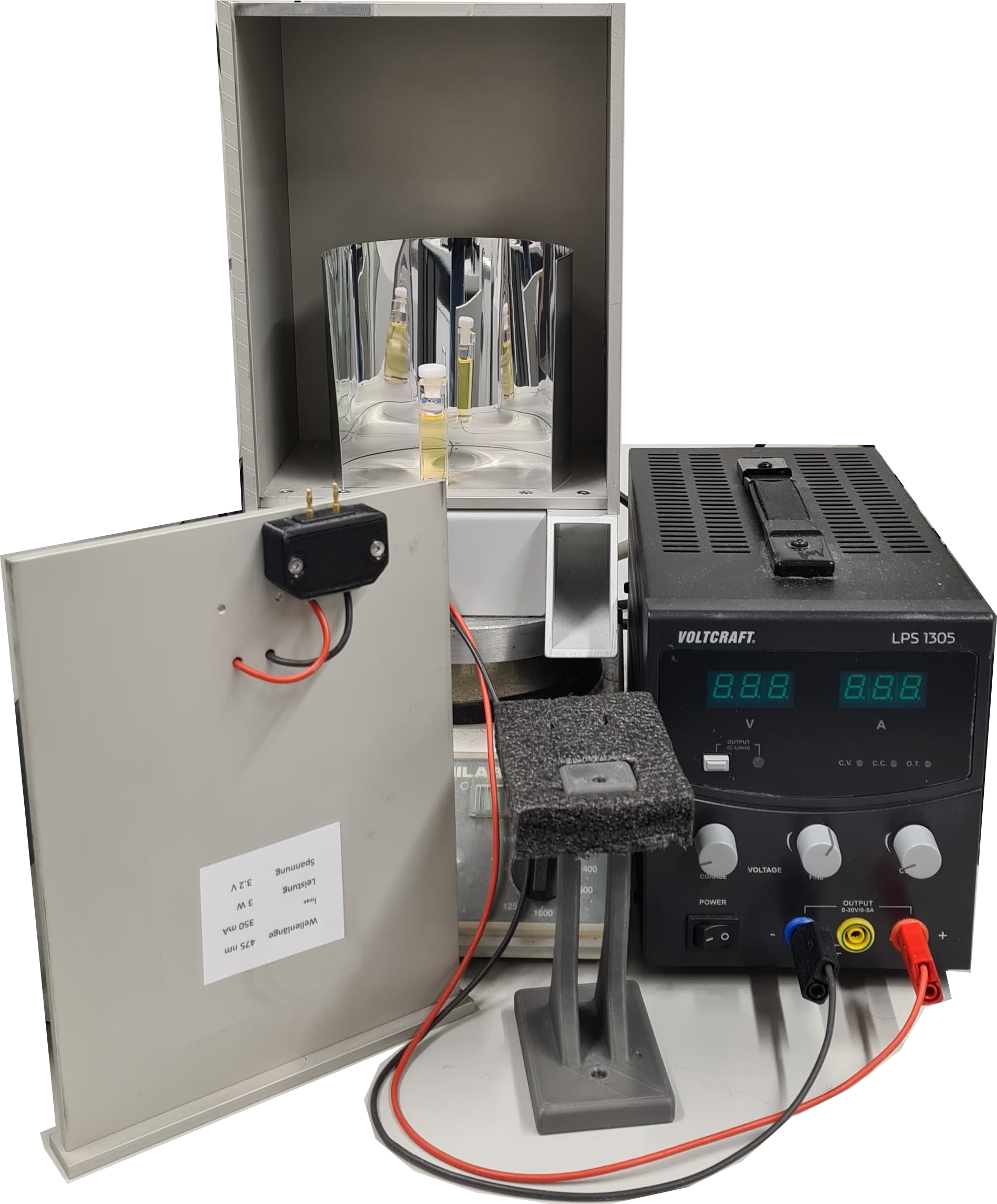


Figure SI 1: Picture of the irradiation chamber used for UV/Vis.

All NMR spectra were recorded on a BRUKER Avance gX spectrometer (^1^H NMR: 300 MHz, 400 MHz, or 500 MHz; ^13^C NMR: 75 MHz, 100 MHz, or 126 MHz). Chemical shifts are given in ppm at room temperature unless stated otherwise and referenced to the residual solvent signal. NBD to QC switching at 310 nm monitored by NMR measurements was performed using the Lucent360^TM^ Advanced Photoreactor by HepatoChem. A 310 nm LED block (approx. maximum power = 50 W) at 80% performance irradiated the NMR tube while cooling the sample to 15 °C. For back-switching at 475 nm, the same setup as for UV/Vis irradiation was applied. Each sample was dissolved in the respective deuterated solvent, transferred to an NMR tube, and flushed with Argon for 30 s before irradiation.

NMR residual signals:

CDCl_3_: δ_H_ = 7.26 ppm δ_C_ = 77.16 ppm

DMSO: δ_H_ = 2.50 ppm δ_C_ = 39.52 ppm

THF-*d_8_*: δ_H_ = 1.72 ppm δ_C_ = 25.31 ppm

Tol-*d_8_*: δ_H_ = 2.09 ppm δ_C_ = 20.43 ppm

MeCN-d_3_: δ_H_ = 1.94 ppm δ_C_ = 1.32, 118.26 ppm

D_2_O: δ_H_ = 4.79 ppm

Syntheses

**General procedures**

A) Purification of NBD **1**: The crude product was dissolved in DCM and washed with saturated NaOH (aq.). The combined aqueous phases were washed with DCM and then acidified by portion wise addition of 2M HCl until neutral. Extraction with DCM after each step and removal of the solvent yielded the product.

B) Synthesis of perylene diimides adapted from literature^[1]^: Imidazole, PTCDA and the respective amine are added under argon to a flame‑dried flask equipped with condenser and heated to 100 °C without stirring. Once an imidazole melt had formed, the reaction was stirred at 100‑130 °C for 3-4 h. After cooling to <100 °C, the solution was neutralized by adding 2M HCl. The precipitate was filtered, washed with H_2_O, and dried under vacuum to give the product in stoichiometric yield.

C) Synthesis of derivatives **12**‑**15** *via* Steglich esterification: NBD **1** and DCC (2 eq) were dissolved in dry DCM (1-5 mL) and cooled to 0 °C. A solution of the precursor alcohol (1 eq) and DMAP (0.5-1.5 eq) in dry DCM (3-15 mL) was added at 0 °C. The mixture was stirred at reflux for 17-52 h. DCM was added and washed with H_2_O and brine. After drying over Na_2_SO_4_ and filtration, the solvent was removed under reduced pressure and the crude product purified by column chromatography (SiO_2_; THF/DCM, 1:99 (v/v)). Further purification was achieved by washing with MeOH and hexane.

**1**:

Route a: Phenylpropiolic acid (505 mg, 3.46 mmol, 1.0 eq) and freshly cracked cyclopentadiene (0.370 mL, 296 mg, 4.48 mmol, 1.3 eq) were dissolved in toluene (4.5 mL) and heated to 110 °C for 16 h under microwave irradiation. The crude product was purified as described in the general procedure and the product was isolated as yellowish-white crystals (287 mg, 1.35 mmol, 39%).

Route b: **2** (94.0 mg, 391 μmol, 1.0 eq) and NaOH (131 mg, 3.28 mmol, 8.4 eq) were dissolved in 3 mL of THF/H_2_O (1:1, v/v) and heated to 100 °C for 21 h. After cooling to room temperature, the mixture was purified as described in the general procedure A). The product was obtained as a white solid (53.3 mg, 251 μmol, 64%). **^1^H NMR** (400 MHz, CDCl_3_): δ [ppm] 10.85 (s, 1H), 7.65-7.56 (m, 2H), 7.47-7.35 (m, 3H), 7.07 (dd, *J* = 5.1, 3.0 Hz, 1H), 6.98 (dd, *J* = 5.1, 3.1 Hz, 1H), 4.16 (s, 1H), 3.93 (s, 1H), 2.34 (d, *J*= 6.7 Hz, 1H), 2.15 (d, *J* = 6.8 Hz, 1H). **^13^C NMR** (101 MHz, CDCl_3_): δ [ppm] 171.1, 169.6, 143.9, 140.7, 138.6, 135.5, 128.9, 128.1, 127.9, 70.6, 59.12, 53.0. **HRMS** (APPI, DCM): calc. for (C_14_H_13_O_2_)^+^: 213.0910, found: *m/z=*213.0911 [M+H]^+^.

**2**:

Ethyl-3-phenylpropiolate (4.34 g, 24.9 mmol, 1.0 eq) and freshly cracked cyclopentadiene (2.61 mL, 2.09 g, 31.7 mmol, 1.3 eq) were heated to 180°C for 3 h under microwave irradiation. The reaction mixture was cooled to room temperature and puriﬁed by distillation over a Vigreux column (80°C, 0.4 mbar). The product was isolated as light-yellow oil (2.73 g, 11.4 mmol, 46%). **^1^H-NMR** (CDCl_3,_ 400 MHz) δ [ppm] = 7.54 – 7.51 (m, 2H), 7.38 – 7.28 (m, 3H), 7.00 – 6.98 (m, 1H), 6.93 – 6.91 (m, 1H), 4.14 (dq, J = 7.1, 0.8 Hz, 2H), 4.08 – 4.05 (m, 1H), 3.87 – 3.84 (m, 1H), 2.26 (dt, J = 6.6, 1.2 Hz, 1H), 2.06 (dt, J = 6.6, 1.4 Hz, 1H), 1.22 (t, J = 7.1 Hz, 3H). **^13^C-NMR** (101 MHz, CDCl_3_) δ [ppm] = 166.48, 165.71, 143.87, 140.98, 139.50, 135.88, 128.61, 127.80, 70.72, 60.24, 58.65, 53.19, 14.28. **HRMS** (APPI, DCM): calc. for (C_16_H_17_O_2_)^+^: 241.1220, found: *m/z* = 241.1223 [M+H]^+^.

**3**:

Following the general procedure B), PTCDA (700 mg, 1.78 mmol, 1 eq) and 6-aminoundecane (762 mg, 4.45 mmol, 2.5 eq) were converted at 130°C for 4 h. The product was obtained as bright red solid in quantitative yield (1.24 g, 1.78 mmol). **^1^H-NMR** (400 MHz, CDCl_3_) δ [ppm] = 8.71 - 8.56 (m, 8H), 5.22 - 5.15 (m, 2H), 2.29-2.20 (m, 4H), 1.91 -1.82 (m, 4H), 1.38 – 1.21 (m, 24H), 0.83 (t, J = 6.7 Hz, 12H).**^13^C-NMR** (101 MHz, CDCl_3_) δ [ppm] = 164.75, 163.69, 134.59, 131.98, 131.23, 129.70, 126.54, 124.06,123.33, 123.12, 54.89, 32.46, 31.89, 26.77, 22.70, 14.18. **HRMS** (MALDI-TOF, DCTB): calc. for (C_46_H_54_N_2_O_4_)^+^: 698.4084, found: *m/z* = 698.4078 [M]^+^.

**4**:

Following the general procedure B), PTCDA (508 mg, 1.29 mmol, 1 eq) and 3‑aminopentane (0.6 mL, 449 mg, 5.05 mmol, 3.9 eq) at 100°C for 3 h yielded the product as bright red solid (634 mg, 1.19 mmol, 92%). **^1^H NMR** (400 MHz, CDCl_3_): δ [ppm] 8.67 (m, 8H), 5.07 (ddd, *J* = 15.4, 9.7, 5.8 Hz, 2H), 2.27 (ddt, *J* = 17.0, 14.8, 7.5, 7.5 Hz, 4H), 1.94 (tt, *J* = 13.4, 13.4, 7.3, 7.3 Hz, 4H), 0.93 (t, *J* = 7.5, 7.5 Hz, 12H). **HRMS** (APPI, DCM): calc. for (C_34_H_31_N_2_O_4_)^+^: 531.2278, found: *m/z=*531.2280 [M+H]^+^.

**5:**

**3** (201 mg, 288 μmol, 1.0 eq) and KOH (81.0 mg, 1.44 mmol, 5.0 eq) were added to a flame-dried flask under Ar atmosphere. The solids were dissolved in *t*‑BuOH (10 mL) and heated under reflux for 2.5 h. The mixture was cooled to room temperature and quenched with AcOH (1.5 mL) and 2M HCl (1 mL). The precipitate was removed by filtration and purified by plug filtration (DCM/AcOH, 99:1). The product was obtained as red solid (75.0 mg, 137 μmol, 48%). **^1^H-NMR** (400 MHz, CDCl_3_) δ [ppm] = 8.73 - 8.66 (m, 8H), 5.22 - 5.15 (m, 1H), 2.29 – 2.19 (m, 2H), 1.91 – 1.82 (m, 2H), 1-37 - 1.21 (m, 12H), 0.83 (t, J = 6.7 Hz, 6H). **^13^C-NMR** (101 MHz, CDCl_3_) δ [ppm] = 160.15, 136.61, 133.75, 124.10, 123.33, 119.20, 55.06, 32.44, 31.87, 26.75, 22.70, 14.18. **HRMS** (MALDI-TOF, DCTB): calc. for (C_35_H_32_NO_5_)^+^: 545.2202, found: *m/z* = 545.2197 [M+H]^+^.

**6**:

**5** (87.0 mg, 159 μmol, 1.0 eq), 6‑aminohexanol (28.8 mg, 246 μmol, 1.5 eq) and imidazole (538 mg) were added to a flask under Ar and heated to 180 °C for 5 h. After cooling to room temperature, EtOH (3 mL) and 2M HCl (15 mL) were added and the mixture stirred for one hour. The solids were removed by filtration and washed with H_2_O before drying under vacuum. The crude product was purified by column chromatography (SiO_2_; DCM/MeOH/AcOH, 99:1:1, (v/v/v)) and trituration with pentane. The product was obtained as a dark red solid (15.5 mg, 24.0 μmol, 15%). **^1^H NMR** (400 MHz, CDCl_3_) δ [ppm] = 8.51-8.19 (m, 8H), 5.21-5.13 (m, 1H), 4.11 (t, 2H), 3.67 (t, J = 7.2 Hz, 2H), 2.30-2.21 (m, 2H), 1.92-1.87 (m, 2H), 1.76-1.73 (m, 2H), 1.64-1.61 (m, 2H), 1.49-1.47 (m, 3H), 1.40-1.24 (m, 14H), 0.85 (t, J = 6.8 Hz, 6H). **^13^C-NMR** (101 MHz, CDCl_3_) δ [ppm] = 163.11, 134.21, 133.90, 131.62, 131.02, 130.87, 129.38, 129.00, 125.97, 122.92, 122.88, 122.76, 62.88, 55.00, 40.53, 32.70, 32.46, 31.93, 28.07, 26.84, 25.42, 22.73, 14.21. **HRMS** (MALDI-TOF, DCTB): calc. for (C_41_H_44_N_2_O_5_)^+^: 644.3250, found: *m/z*=644.3245 [M+H]^+^.

**7**:

**5** (75.0 mg, 137 μmol, 1.0 eq), 4‑aminophenol (28.0 mg, 257 μmol, 1.9 eq) and imidazole (495 mg) were combined and heated to 180°C for 4 h. After cooling to room temperature, EtOH (3 mL) and 2M HCl (15 mL) were added and the mixture stirred for one hour. The precipitate was filtered and washed with H_2_O. The crude product was purified by column chromatography (SiO_2_; DCM/MeOH/AcOH, 98:1:1, v/v/v) and obtained as a purplish-red solid (64.4 mg, 101 μmol, 74%). **^1^H NMR** (500 MHz, C_2_D_2_Cl_4_, 90°C) δ [ppm] = 8.67-8.59 (m, 8H), 7.17 (d, J = 8.7 Hz, 2H), 6.96 (d, J = 8.7 Hz, 2H), 5.15-5.09 (m, 1H), 2.23-2.14 (m, 2H), 1.90-1.83 (m, 2H), 1.33-1.24 (s, 12H), 0.82 (t, J = 6.8 Hz, 6H). **^13^C-NMR** (126 MHz, C_2_D_2_Cl_4_, 90°C) δ [ppm] = 163.82, 156.01, 135.50, 134.61, 132.02, 131.70, 130.24, 130.15, 129.83, 128.24, 127.07, 126.89, 126.79, 123.74, 123.56, 123.30, 120.63, 116.48, 99.92, 55.19, 32.71, 31.92, 26.83, 22.67, 14.12. **HRMS** (MALDI-TOF, DCTB): calc. for (C_41_H_37_N_2_O_5_)^+^: 637.2702, found: *m/z*=637.2697 [M+H]^+^.

**8**:

**4** (508 mg, 957 μmol, 1 eq), B_2_pin_2_ (327 mg, 1.29 mmol, 1.3 eq), Ir[(OMe)cod]_2_ (30.2 mg, 46.0 μmol, 0.05 eq), and P(C_6_F_5_)_3_ (61.5 mg, 116 μmol, 0.1 eq) were added under inert gas to a flame‑dried flask and dissolved in deoxygenated, dry 1,4-dioxane (6 mL). The reaction was heated to 110 °C for 70 h. After cooling to room temperature and removing the solvent under vacuum, the crude product was filtered through a short silica plug eluted with DCM. The filtrate was concentrated under reduced pressure. The residue was washed with MeOH and recrystallized from DCM/MeCN. The product was obtained as dark red solid (350 mg, 0.533 mmol, 56%). **^1^H NMR** (400 MHz, CDCl_3_) δ [ppm] 8.70 – 8.58 (m, 6H), 8.54 (m, 1H), 5.13 – 5.03 (m, 1H), 5.03 – 4.94 (m, 1H), 2.35 – 2.16 (m, 4H), 1.94 (m, 4H), 0.97 – 0.88 (m, 12H). **HRMS** (APPI, DCM): calc. for (C_40_H_42_BN_2_O_6_)^+^: 657.3130, found: *m/z=*657.3149 [M+H]^+^.

**9**:

**8** (208 mg, 317 μmol, 1 eq), NaOH (122 mg, 3.05 mmol, 20 eq), and hydroxylammonium chloride (142 mg, 2.04 mmol, 13 eq) were dissolved in EtOH (20 mL). The reaction was stirred at room temperature for 18 h. The solution was acidified with 2M HCl, neutralized with saturated NaHCO_3_ (aq.), and extracted with DCM. The combined organic phases were washed with H_2_O, dried over Na_2_SO_4_, filtered, and the solvent removed under reduced pressure. The crude product was purified by column chromatography (SiO_2_; THF/DCM, 1:99 (v/v)). The product was isolated as bright red solid (144 mg, 263 μmol, 83%). **^1^H NMR** (400 MHz, CDCl_3_): δ [ppm] 13.21 (s, 1H), 8.62-8.49 (m, 3H), 8.34 (dd, *J* = 18.8, 8.1 Hz, 2H), 8.21 (d, *J* = 8.3 Hz, 1H), 7.79 (s, 1H), 5.06 (tt, *J* = 9.5, 9.5, 5.9, 5.9 Hz, 2H), 2.35-2.19 (m, 4H), 1.98 (m, 4H), 0.96 (td, *J* = 7.5, 7.4, 1.0 Hz, 12H). **^13^C NMR** (101 MHz, CDCl_3_) δ [ppm] 134.7, 134.2, 133.7, 130.6, 126.4, 123.7, 123.4, 121.2, 120.6, 115.6, 57.9, 25.2, 25.1, 11.5, 11.5. **HRMS** (APPI, DCM): calc. for (C_34_H_31_N_2_O_5_)^+^: 547.2227, found: *m/z=*547.2241 [M+H]^+^.

**10**:

To a flame-dried pressure vial, **8** (96 mg, 146 μmol, 1 eq) and CuBr_2_ (148 mg, 662 μmol, 4.5 eq) were added under Ar atmosphere. The solids were dissolved in 1,4‑dioxane, MeOH, and H_2_O (5:2:1, v/v/v). The solution was saturated with Ar for 5 min and heated to 120 °C for 18 h. After cooling to room temperature, the mixture was poured onto H_2_O (50 mL) and extracted with DCM. The combined organic phases were dried over Na_2_SO_4_, filtered, and the solvent removed under reduced pressure. After purification by column chromatography (1:199 THF/DCM (v/v)), the product was isolated as bright red solid (60 mg, 99 μmol, 68%). **^1^H NMR** (400 MHz, CDCl_3_) δ [ppm] 8.79 – 8.51 (m, 7H), 5.07 (ttd, *J* = 9.4, 9.4, 5.9, 5.8, 3.0 Hz, 2H), 2.26 (m, 4H), 2.04 – 1.89 (m, 4H), 0.94 (td, *J* = 7.5, 7.4, 1.1 Hz, 12H). **HRMS** (APPI, DCM): calc. for (C_34_H_30_BrN_2_O_4_)^+^: 609.1383, found: *m/z=*609.1393 [M+H]^+^.

**11**:

**10** (30.0 mg, 49.2 μmol, 1 eq), CuI (4.2 mg, 22 μmol, 0.4 eq), Pd(PPh_3_)_4_ (6.2 mg, 5.4 μmol, 0.1 eq), and 3-butyn-1-ol (10 μL, 9.3 mg, 132 μmol, 2.7 eq) were dissolved in THF (2 mL). The solution was saturated with Ar for 5 min, NEt_3_ (1 mL) added and stirred for 5 h at room temperature. DCM (50 mL) was added and the solution washed with brine. The combined aqueous phases were extracted with DCM. The organic phases were combined, dried over Na_2_SO_4_, filtered and the solvent removed under reduced pressure. The crude product was purified by column chromatography (SiO_2_; THF/DCM, 1:99 (v/v)). The product was obtained as deep red-violet solid (18 mg, 30 μmol, 61%). **^1^H NMR** (400 MHz, CDCl_3_) δ [ppm] 8.74-8.64 (m, 3H), 8.63-8.55 (m, 4H), 5.07 (m, 2H), 3.98 (m, 2H), 2.89 (t, *J* = 5.4, 5.4 Hz, 2H), 2.35-2.17 (m, 4H), 1.95 (m, 4H), 0.93 (td, *J* = 7.5, 7.5, 2.9 Hz, 12H). **^13^C NMR** (101 MHz, CDCl_3_) δ [ppm] 134.2, 133.5, 130.1, 129.3, 128.2, 126.2, 125.4, 123.1, 123.0, 122.9, 60.8, 57.6, 24.9, 24.9, 11.3, 11.2. **HRMS** (APPI, DCM): calc. for (C_38_H_35_N_2_O_5_)^+^: 599.2540, found: *m/z=*599.2555 [M+H]^+^.

**12**:

According to the general procedure C), **1** (28.6 mg, 135 μmol, 2.4 eq) and **6** (37.0 mg, 57.4 μmol, 1.0 eq) were converted. The product was obtained as a dark red solid (39.6 mg, 47.2 μmol, 82%).**^1^H NMR** (500 MHz, THF-*d_8_*) δ [ppm] = 8.50-8.27 (m, 8H), 7.59-7.54 (m, 2H), 7.34-7.28 (m, 2H), 7.28-7.24 (m, 1H), 6.98-6.95 (s, 1H), 6.95-6.93 (s, 1H), 5.25-5.17 (m, 1H), 4.12-4.01 (m,5H), 3.84 (s, 1H), 2.40-2.31 (m, 2H), 2.19 (dt, J = 6.5, 1.5 Hz, 1H), 2.00 (dt, J = 6.5, 1.5 Hz, 1H),1.93-1.85 (m, 2H), 1.73 (m, 2H,), 1.63 (p, J = 6.8 Hz, 2H), 1.46-1.27 (m,16H), 0.88 (t, J = 6.9 Hz, 6H).**^13^C-NMR** (126 MHz, THF-*d_8_*) δ [ppm] = 171.33, 170.63, 163.29, 162.02, 161.08, 144.17, 141.57,140.80,140.19, 138.30, 134.73, 131.22, 131.13, 129.97, 129.59, 129.49, 128.64, 128.19,128.10, 126.61, 126.55, 123.98, 123.88, 123.84, 64.23, 54.90, 40.77, 37.30, 33.81, 33.19,33.12, 32.72, 32.28, 32.08, 30.52, 29.44, 28.81, 28.70, 27.64, 27.45, 26.57, 23.38, 21.27,14.34. **HRMS** (MALDI‑TOF, DCTB): calc. for (C_55_H_54_N_2_O_6_)^+^: 838.3982, found: *m/z*=838.3976 [M+H]^+^. **UV/Vis** (THF): λ_max_ (ε [L*mol^‑1^*cm^-1^]) = 522 nm (77400), 486 nm (46900), 455 nm (17000). **Fluorescence** (THF, exc. 486 nm): l_max_ (rel. Int.) = 531 (100%), 570 (40%), 614 (6%) nm.

**13**:

According to the general procedure C), **1** (12 mg, 57 μmol, 1.5 eq) and **7** (24 mg, 38 μmol, 1.0 eq) were converted. The product was obtained as a dark red solid (12 mg, 14 μmol, 37%). **^1^H NMR** (500 MHz, THF-*d_8_*) δ [ppm] = 8.55-8.45 (m, 8H), 7.72-7.69 (m, 2H), 7.41-7.37 (m, 4H), 7.34-7.30 (m, 1H), 7.28-7.25 (m, 2H), 7.12-7.10 (m, 1H), 7.06-7.04 (m, 1H), 5.22-5.16 (m, 1H), 4.26 (s, 1H), 3.99 (s, 1H), 2.36-2.34 (m, 1H), 2.34-2.29 (m, 2H), 2.14 (dt, J = 6.6,1.4 Hz, 1H), 1.92-1.86 (m, 2H), 1.40-1.27 (m, 12H), 0.86 (t, J = 7.0 Hz, 6H). **^13^C-NMR** (126 MHz, THF-*d_8_*) δ [ppm] = 169.32, 163.45, 163.29, 151.62, 144.30, 141.65, 139.20,136.43, 135.07, 134.84, 133.52, 131.39, 130.67, 130.04, 129.93, 129.45, 128.85, 128.39,126.84, 126.74, 124.39, 124.11, 124.09, 122.52, 70.92, 59.61, 54.93, 54.22, 33.09, 32.65,30.52, 27.48, 23.35, 14.31. **HRMS** (MALDI-TOF, DCTB): calc. for (C_55_H_46_N_2_NaO_6_)^+^: 853.3259, found: *m/z*=853.3248 [M+Na]^+^. **UV/Vis** (THF): λ_max_  (ε [L*mol^-1^*cm^-1^]) = 521 nm (93000), 486 nm (56300), 455 nm (20600). **Fluorescence** (THF, exc. 486 nm): l_max_ (rel. Int.) = 531 (100%), 570 (38%), 612 (6%) nm.

**14**:

According to the general procedure C), **1** (146 mg, 688 μmol, 3.8 eq) and **9** (101 mg, 185 μmol, 1 eq) were converted. The product was isolated as bright red solid (30.0 mg, 40.5 μmol, 22%). **^1^H NMR** (500 MHz, CDCl_3_): δ [ppm] 8.71-8.58 (m, 5H), 8.48 (m, 1H), 8.15 (s, 1H), 7.77-7.70 (m, 2H), 7.39-7.29 (m, 3H), 7.21 (m, 1H), 7.06 (m, 1H), 5.11-4.98 (m, 2H), 4.49-4.43 (m, 1H), 4.03 (m, 1H), 2.55 (m, 1H), 2.29-2.21 (m, 5H), 1.96-1.89 (m, 4H), 0.92 (m, 12H). **^13^C NMR** (126 MHz, CDCl_3_) δ [ppm] 144.2, 140.6, 138.1, 135.3, 134.4, 134.3, 133.6, 131.0, 129.5, 129.1, 128.2, 127.8, 123.5, 123.3, 122.5, 120.7, 70.8, 58.9, 57.7, 53.5, 25.0, 25.0, 24.9, 11.3. **HRMS** (APPI, DCM): calc. for (C_48_H_41_N_2_O_6_)^+^: 741.2959, found: *m/z=*741.2949 [M+H]^+^. **UV/Vis** (THF): λ_max_ (ε [L*mol^‑1^*cm^-1^]) = 520 nm (86700), 485 nm (56000), 454 nm (21400). **Fluorescence** (THF, exc. 465 nm): l_max_ (rel. Int.) = 542 (96%), 576 (100%), 627 (29%) nm.

**15**:

According to the general procedure C), **1** (7.7 mg, 36 μmol, 1.2 eq) and **11** (18 mg, 30 μmol, 1.0 eq) were converted. The product was obtained as pinkish-red solid (5.0 mg, 6.3 μmol, 21%). **^1^H NMR** (400 MHz, CDCl_3_): δ [ppm] 8.72-8.57 (m, 7H), 7.61-7.55 (m, 2H), 7.39-7.27 (m, 3H), 7.01 (m, 1H), 6.92 (m, 1H), 5.07 (tt, *J* = 9.5, 9.5, 5.9, 5.9 Hz, 2H), 4.48 (t, *J* = 6.8, 6.8 Hz, 2H), 4.19 (s, 1H), 3.88 (s, 1H), 3.01 3.01 (t, *J* = 6.7, 6.7 Hz, 2H), 2.35-2.18 (m, 5H), 2.13-2.05 (m, 1H), 2.02-1.87 (m, 4H), 0.93 (td, *J* = 7.5, 7.4, 3.0 Hz, 12H). **^13^C NMR** (101 MHz, CDCl_3_) δ [ppm] 143.6, 140.7, 135.4, 134.0, 133.6, 129.7, 128.6, 127.7, 127.6, 123.1, 122.9, 70.4, 61.6, 58.5, 57.6, 52.9, 24.9, 24.8, 20.9, 11.3, **HRMS** (APPI, DCM): calc. for (C_52_H_45_N_2_O_6_)^+^: 793.3272, found: *m/z=*793.3248 [M+H]^+^. **UV/Vis** (THF): λ_max_ (ε [L*mol^‑1^*cm^‑1^]) = 521 nm (63500), 485 nm (38800), 454 nm (14700). **Fluorescence** (THF, exc. 465 nm): l_max_ (rel. Int.) = 538 (95%), 573 (100%), 623 (23%) nm.

**Cobalt porphyrin catalyst Por:**

For the QC‑to‑NBD isomerization of pristine **1**, the following catalyst was used which was synthesized *via* literature procedure.^[2]^

**^1^H-NMR** (300 MHz, CDCl_3_) δ [ppm] = 8.94 – 8.85 (m, 6H), 8.78 (d, 3J = 4.5 Hz, 2H), 8.51 (d, 3J = 8.0 Hz, 2H), 8.36 (d, 3J = 8.1 Hz, 2H), 8.15 (d, 3J = 8.0 Hz, 6H), 7.77 (d, 3J = 8.0 Hz, 6H), 1.62 (s, 27H), -2.75 (s, 2H).

Cobalt- 5-(p-Carboxymethylphenyl)-10,15,20-(p-tert-butyltriphenylphenyl)porphyrin (**Por**) :

**HRMS** (MALDI,DCTB): calculated *m/z* = 883.3417, measured *m/z* = 883.3428 [M^+^].

NMR spectra

Figure SI 2: ^1^H NMR spectrum of **1** in CDCl_3_.

Figure SI 3: ^13^C NMR spectrum of **1** in CDCl_3_.

Figure SI 4: ^1^H NMR spectrum of the QC isomer of **1** in CDCl_3_.

Figure SI 5: ^13^C NMR spectrum of the QC isomer of **1** in CDCl_3_.

Figure SI 6: ^1^H NMR spectrum of **6** in CDCl_3_.

Figure SI 7: ^13^C NMR spectrum of **6** in CDCl_3_.

Figure SI 8: ^1^H NMR spectrum of **7** in in CD_2_Cl_2_ at 90 °C.

Figure SI 9: ^13^C NMR spectrum of **7** in CD_2_Cl_2_ at 90 °C.

Figure SI 10: ^1^H NMR spectrum of **9** in CDCl_3_.

Figure SI 11: ^13^C NMR spectrum of **9** in CDCl_3_.

Figure SI 12: ^1^H NMR spectrum of **11** in CDCl_3_.

Figure SI 13: ^13^C NMR spectrum of **11** in CDCl_3_.


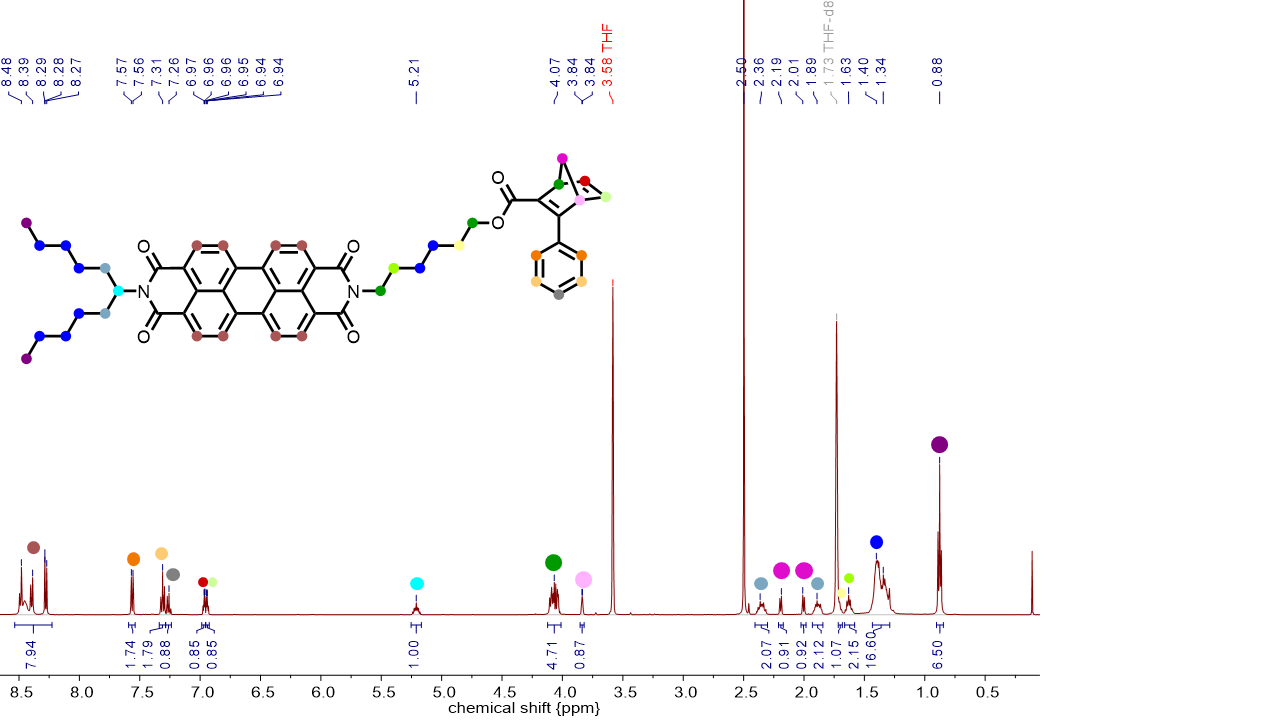


Figure SI 14: ^1^H NMR spectrum of **12** in THF‑d_8_. The proton assignment was performed using 2D NMR methods and is indicated by colors.

Figure SI 15: ^13^C NMR spectrum of **12** in THF‑d_8_.

Figure SI 16: COSY NMR spectrum of **12** in THF‑d_8_.

Figure SI 17: HSQC NMR spectrum of **12** in CDCl_3_.


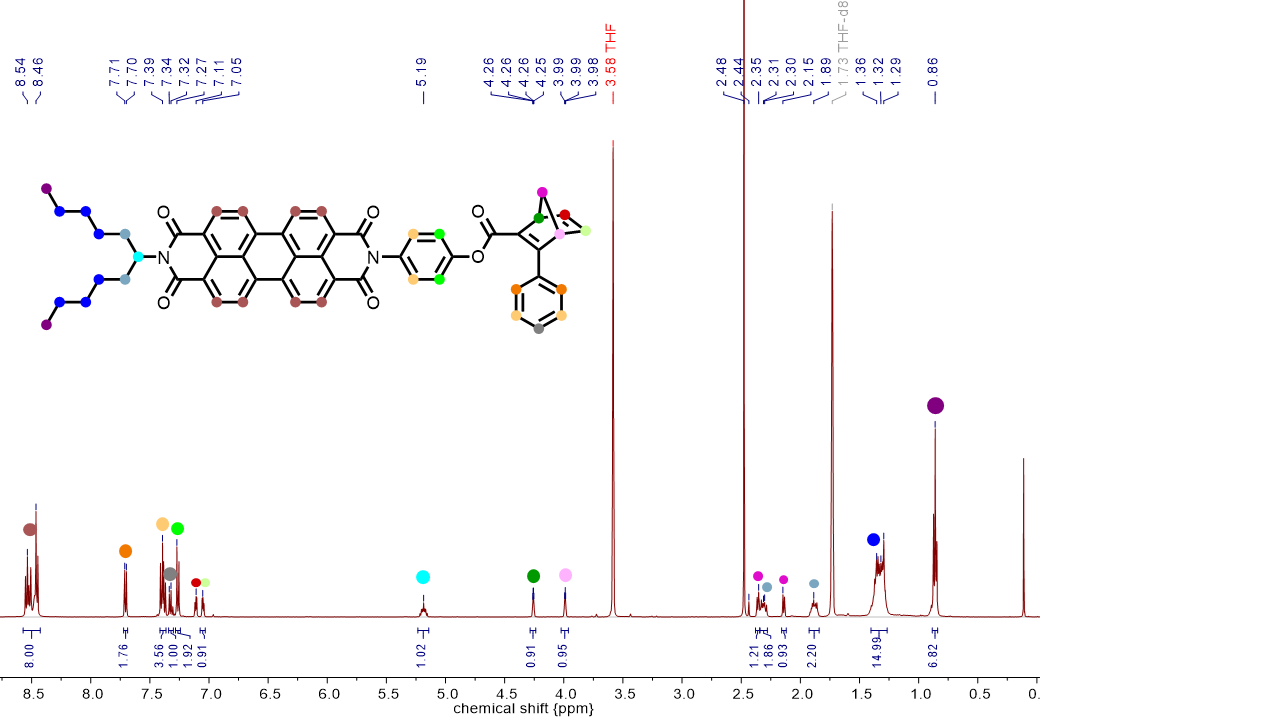


Figure SI 18: ^1^H NMR spectrum of **13** in THF‑d_8_. The proton assignment was performed using 2D NMR methods and is indicated by colors.

Figure SI 19: ^13^C NMR spectrum of **13** in THF‑d_8_.

Figure SI 20: COSY NMR spectrum of **13** in THF‑d_8_.

Figure SI 21: HSQC NMR spectrum of **13** in CDCl_3_.


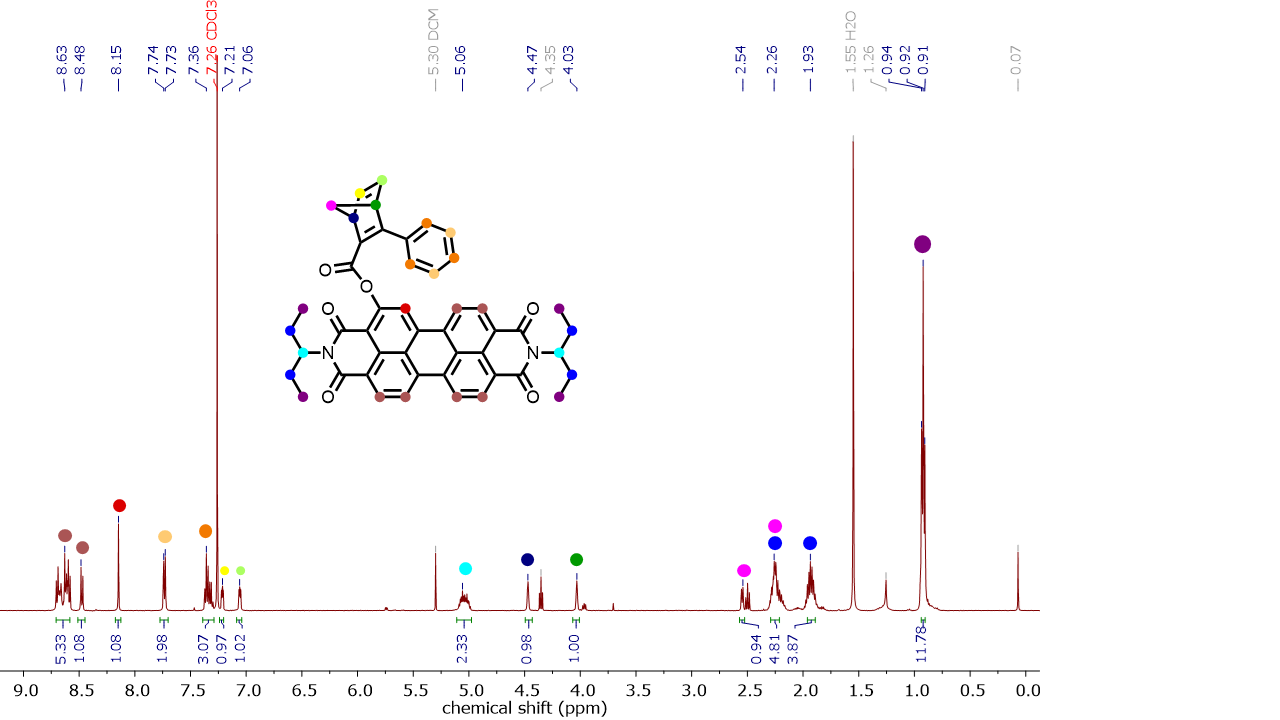


Figure SI 22: ^1^H NMR spectrum of **14** in CDCl_3_. The proton assignment was performed using 2D NMR methods and is indicated by colors.

Figure SI 23: ^13^C NMR spectrum of **14** in CDCl_3_.

Figure SI 24: COSY NMR spectrum of **14** in CDCl_3_.

Figure SI 25: HSQC NMR spectrum of **14** in CDCl_3_.


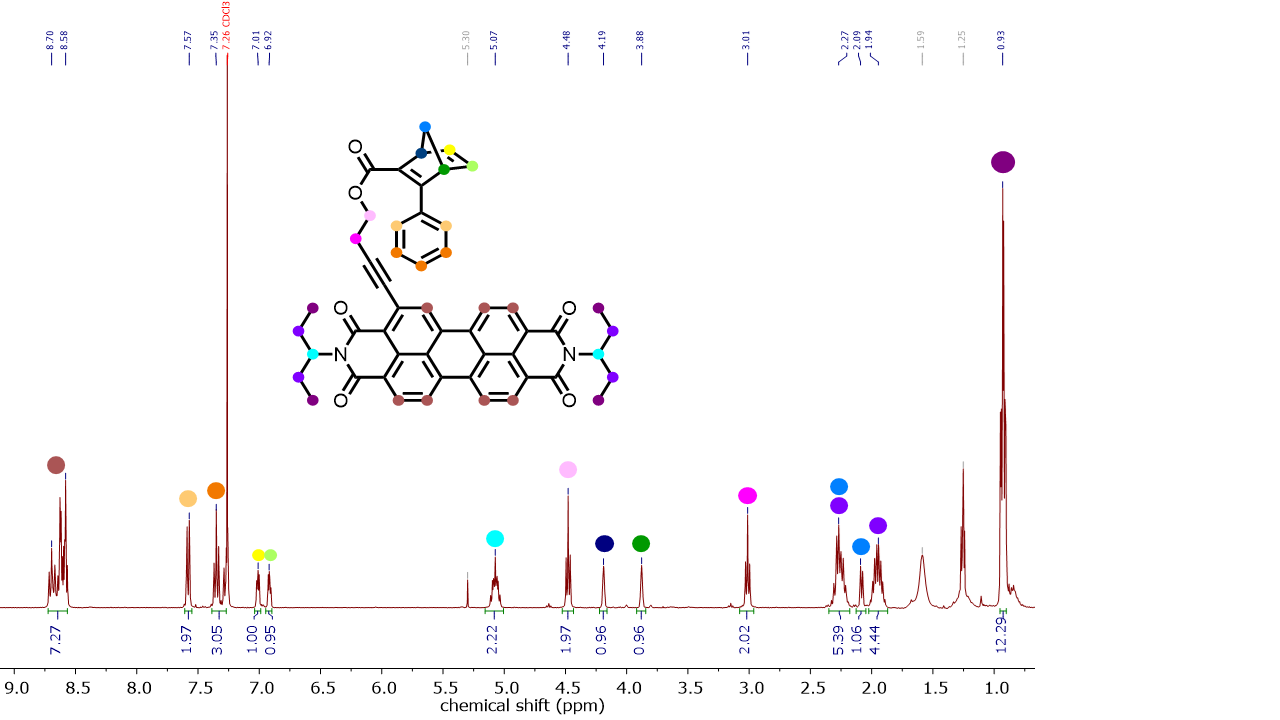


Figure SI 26: ^1^H NMR spectrum of **15** in CDCl_3_. The proton assignment was performed using 2D NMR methods and is indicated by colors.

Figure SI 27: ^13^C NMR spectrum of **15** in CDCl_3_.

Figure SI 28: COSY NMR spectrum of **15** in Tol‑d_8_.

Figure SI 29: HSQC NMR spectrum of **15** in CDCl_3_.

Fluorescence quenching study

It is noteworthy that the overall solubility of PDIs is significantly higher in chlorinated solvents which allowed for higher concentrations and thus more fluorescence appearing in photographs.

**
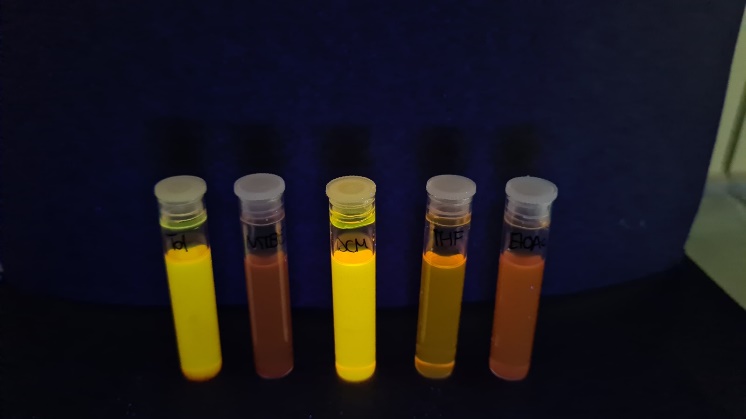
**

Figure SI 30: Fluorescence spectra of 2.5 mM solutions of **7** in solvents of different polarity after excitation at 486 nm (top). Photographs of visible fluorescent quenching under UV light (bottom) in the order from left to right: Tol-MTBE-DCM-THF-EtOAc. The increasing solvent polarity based on their dipole moment: Toluene (Tol); DCM; Methyl tert‑butyl ether (MTBE); THF; Ethyl acetate (EtOAc).

Absorption and emission data

Figure SI 31: Extinction coefficient (solid lines) and fluorescence spectra (dashed lines) of the precursor (left) and target compounds (right). All spectra were recorded in THF except for the fluorescence spectrum of **7** which was recorded in toluene.

|  | $\boldsymbol{\lambda(}\boldsymbol{\varepsilon}_{\boldsymbol{max}}\boldsymbol{)}$ (THF) | $\boldsymbol{\varepsilon}_{\boldsymbol{max}}$ (THF) [L*mol^-1^*cm^-1^] | $\boldsymbol{\lambda(}{\boldsymbol{fl}\boldsymbol{uo}}_{\boldsymbol{max}}\boldsymbol{)}$ (THF, Tol*) |
| --- | --- | --- | --- |
| $\boldsymbol{\lambda}_{\boldsymbol{exc}}$ = 486 nm |  |  |  |
| Precursor 6 | 521, 486, 455 nm | 84400, 51100, 18600 | 530, 570, 614 nm |
| Target molecule 12 | 522, 486, 455 nm | 77400, 46900, 17000 | 531, 570, 614 nm |
| Precursor 7 | 521, 486, 455 nm | 102900, 62000, 22600 | 536, 577, 629 nm* |
| Target molecule 13 | 521, 486, 455 nm | 93000, 56300, 20600 | 531, 570, 612 nm |
| $\boldsymbol{\lambda}_{\boldsymbol{exc}}$ = 465 nm |  |  |  |
| Precursor 9 | 528, 492, 462 nm | 71344, 49231, 20634 | 535, 576, 626 nm |
| Target molecule 14 | 520, 485, 455 nm | 86722, 55980, 21365 | 542, 576, 627 nm |
| Precursor 11 | 522, 486, 455 nm | 47427, 29365, 11386 | 531, 570, 611 nm |
| Target molecule 15 | 521, 485, 454 nm | 63476, 38789, 14667 | 538, 573, 623 nm |

Table SI 1: Summary of the extinction coefficient values at the maxima ($\varepsilon_{max}$) and the respective wavelengths $\lambda(\varepsilon_{max}$) where they are located and the wavelengths at which maximum fluorescence is observed ($\lambda({fluo}_{max})$.

Table SI 2: Stokes shifts of all compounds.

| Precursors | Stokes shift | Target molecules | Stokes shift |
| --- | --- | --- | --- |
| 6 | 9 nm | **12** | 9 nm |
| 7 | 15 nm | **13** | 10 nm |
| 9 | 12 nm | **14** | 22 nm |
| 11 | 9 nm | **15** | 17 nm |

UV/Vis assays

The irradiation studies were used for semi‑quantitative comparison of conversion yields. That is due to the superimposition of PDI‑ and NBD‑centered absorption which prohibits the determination of full conversion to QC based on a vanishing NBD absorption. The calculation approach for those yields is exemplified in **Figure SI 32**. Hereby, the initial NBD absorption intensity (blue line) is assigned to only NBD isomer being present. After a consistent trajectory has settled upon prolonged irradiation at 310 nm, complete conversion to QC is assumed (red line). The difference between the max. and min. absorption (X) at one wavelength (300 or 310 nm were used) is defined as the total absorption frame in which isomerization ratios can be tracked. The difference (Y) between the absorption after maximal back‑conversion and the minimal NBD absorption is then divided by X to give the semi‑quantitative, approximate ratio of regenerated NBD (N).

$$N=\frac{Y}{X}= \frac{0.07371-0.04493}{0,1169-0,04493}=40\%$$


Figure SI 32: Illustration of the calculation procedure for the determination of back‑conversion yields in UV/Vis assays.

Interconversion 12 and 16:

Figure SI 33: Irradiation study of **12** in THF, monitored by UV/Vis.

Figure SI 34: Irradiation study of **12** in Tol, monitored by UV/Vis.

**Interconversion 13 and 17:**

Figure SI 35: Irradiation study of **13** in THF, monitored by UV/Vis.

Figure SI 36: Irradiation study of **13** in Tol, monitored by UV/Vis.

Solvent dependency investigation of back‑switch efficiency of **17**:

Figure SI 37: Solvent‑dependent irradiation study of **17**  to **13** in DCM and EtOAc, monitored by UV/Vis.

Interconversion 14 and 18:

Figure SI 38: Irradiation study of **14** in Tol, monitored by UV/Vis.

Interconversion 15 and 19:

Figure SI 39: Irradiation study of **15** in THF, monitored by UV/Vis.

Figure SI 40: Irradiation study of **15** in Tol, monitored by UV/Vis.

Interconversion studies of 1:

Figure SI 41: Irradiation study of **1** in H_2_O (left) and acetonitrile MeCN (right), monitored by UV/Vis. Back‑conversion was achieved by addition of the cobalt porphyrin catalyst **Por**. The pH value was adjusted by addition of a concentrated NaOH and 2 M HCl solution to a cuvette containing a neutral aqueous solution of **1**, respectively.

Figure SI 42: Irradiation study of **1** in THF.

Reference assays of unlinked NBD 1 and PDI precursor 6

Reference NMR irradiation assay of unlinked **1** as confirmation of intermolecularly occurring back‑isomerization. A THF‑*d_8_* solution containing **1** and **6** was irradiated similar to the dyads before and the interconversion observed over time. Noticeable back‑switching to NBD confirms that no covalent linkage of the photoactive catalyst to NBD is required to introduce back‑isomerization.

Figure SI 43: Time progression of the interconversion of pristine **1** in a solution containing precursor **6** (8 mM both) upon irradiation at 310 nm (left) and 475 nm (right) in THF‑d_8_ compared to covalently imide-linked **12** and **13**.

Similar experiments were performed in low concentration UV/Vis irradiation studies:

Figure SI 44: Irradiation study of unlinked **1** and **6** in THF (top) and Tol (bottom), monitored by UV/Vis.

NMR assays

The signals indicating ongoing interconversion are highlighted by the boxes.


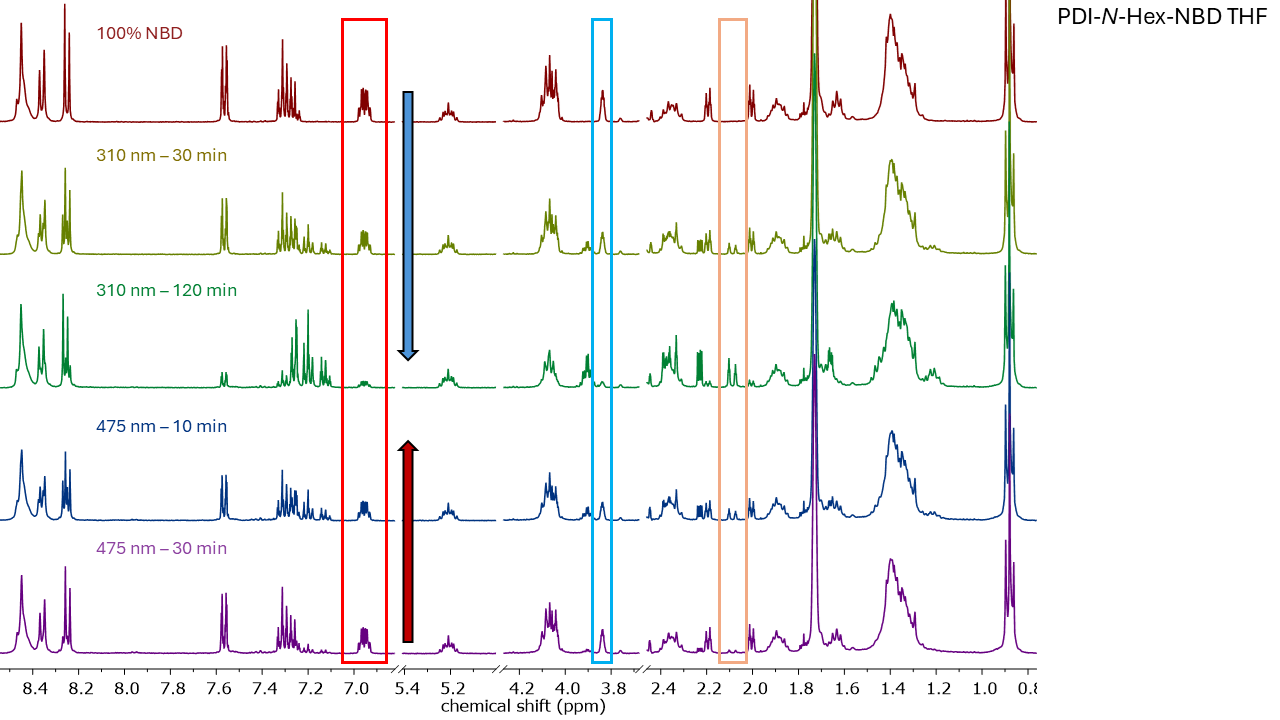


Figure SI 45: Interconversion of **12** in **THF-d_8_**. Red=NBD olefinic proton signals; blue=NBD bridgehead proton signals; orange=QC signals.


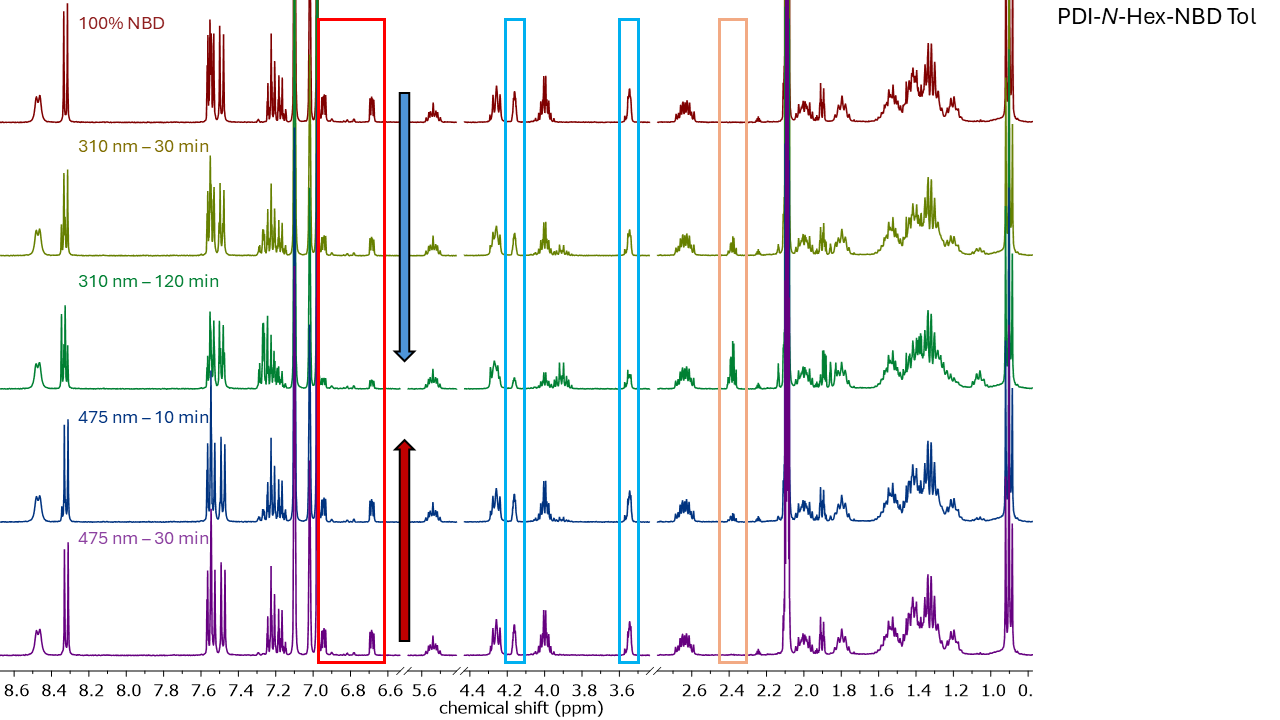


Figure SI 46: Interconversion of **12** in **Tol-d_8_**. Red=NBD olefinic proton signals; blue=NBD bridgehead proton signals; orange=QC signals.


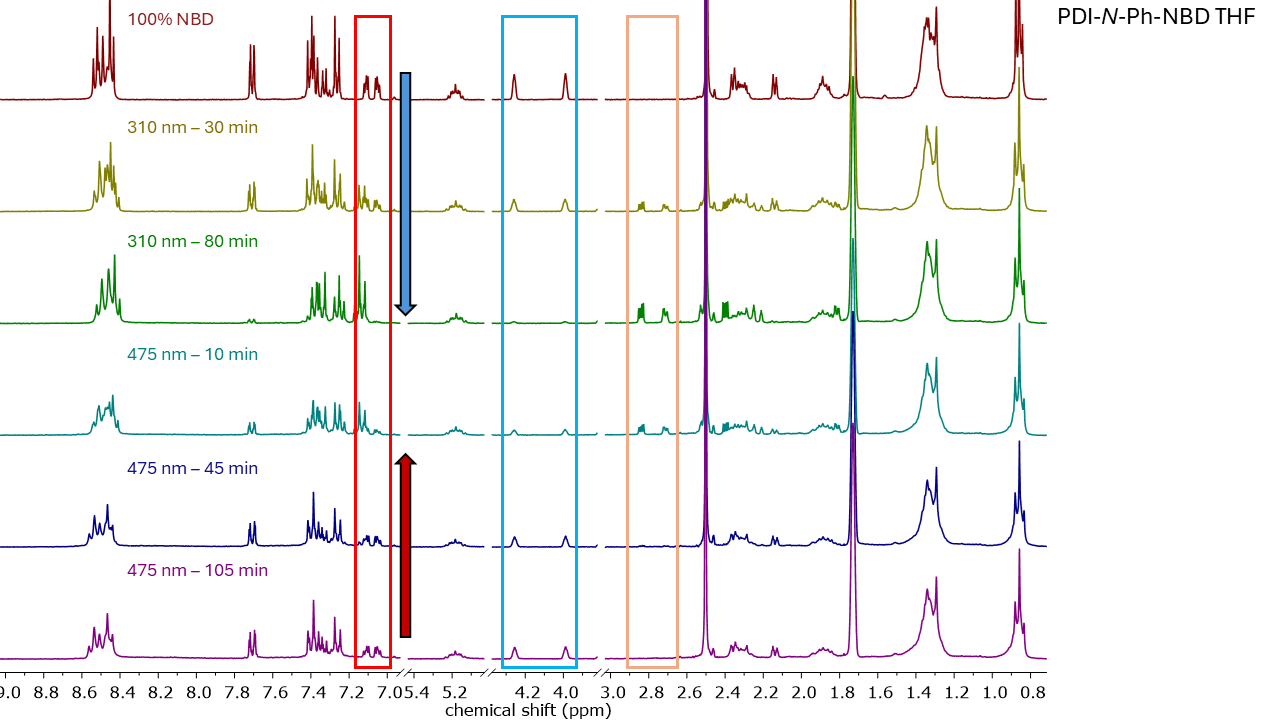


Figure SI 47: Interconversion of **13** in **THF-d_8_**. Red=NBD olefinic proton signals; blue=NBD bridgehead proton signals; orange=QC signals.


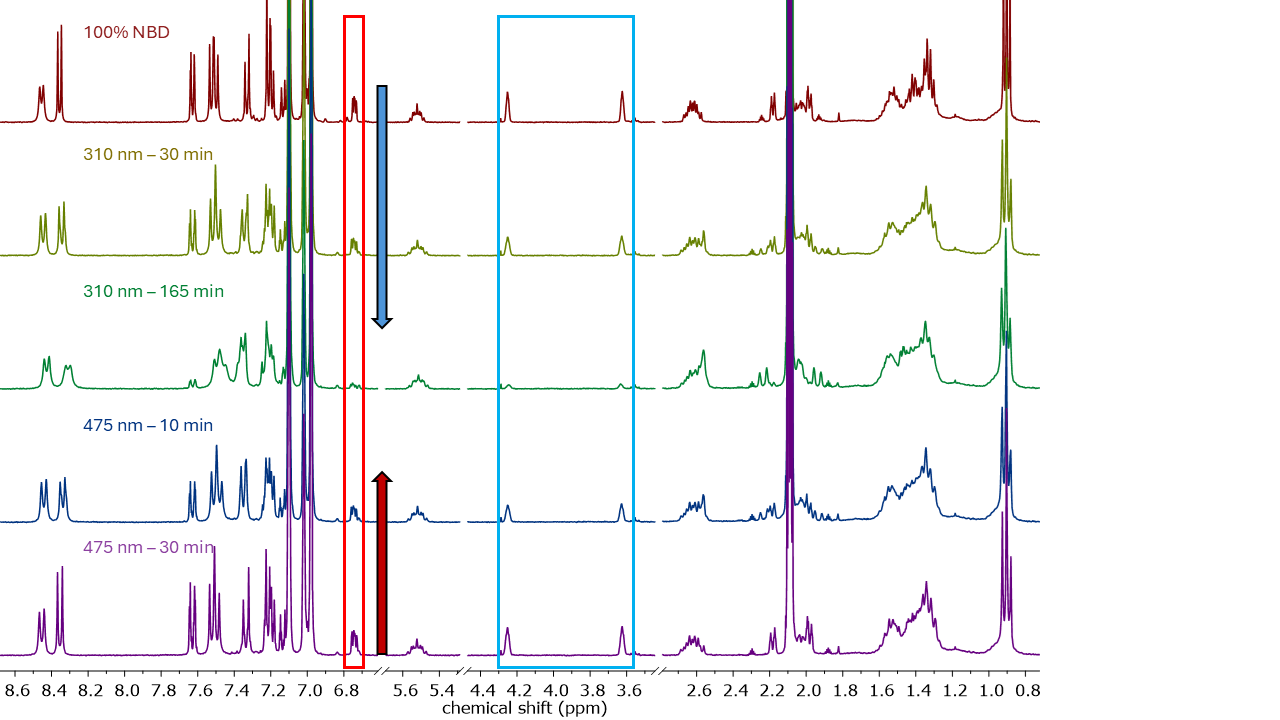


Figure SI 48: Interconversion of **13** in **Tol-d_8_**. Red=NBD olefinic proton signals; blue=NBD bridgehead proton signals.


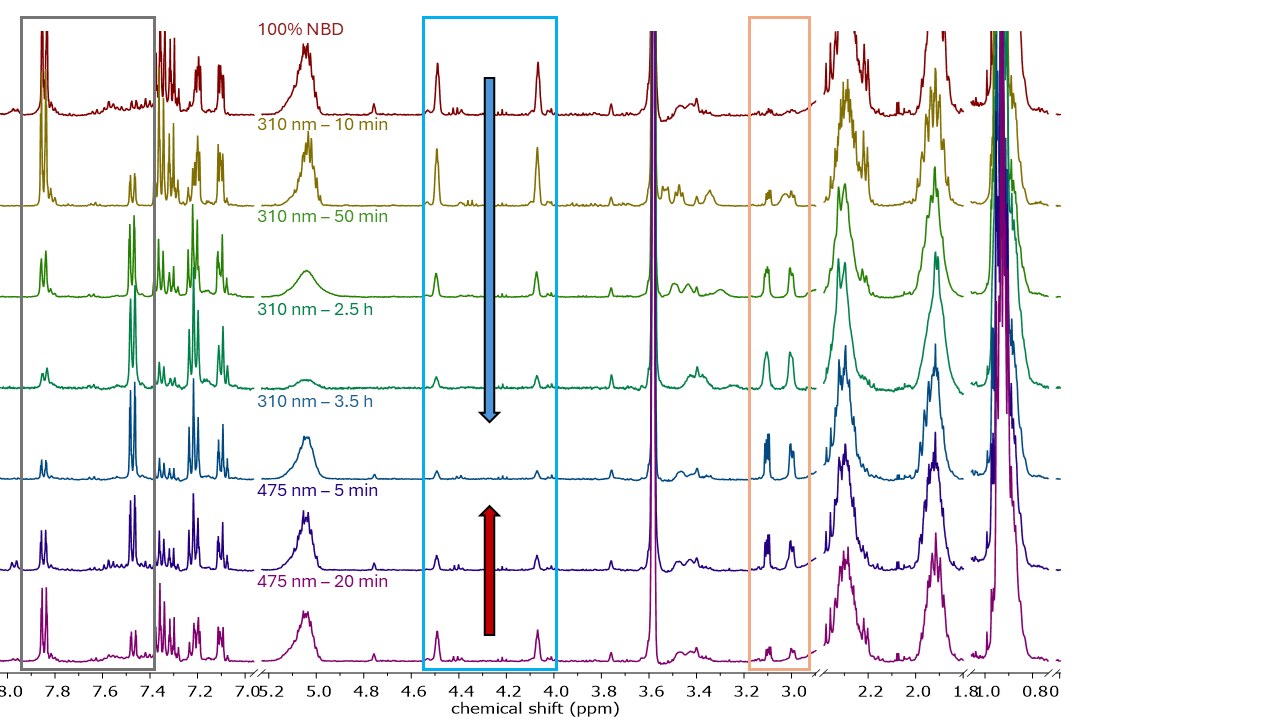


Figure SI 49: Interconversion of **14** in **THF-d_8_**. Blue=NBD bridgehead proton signals; grey=phenyl proton signals; orange=QC signals.


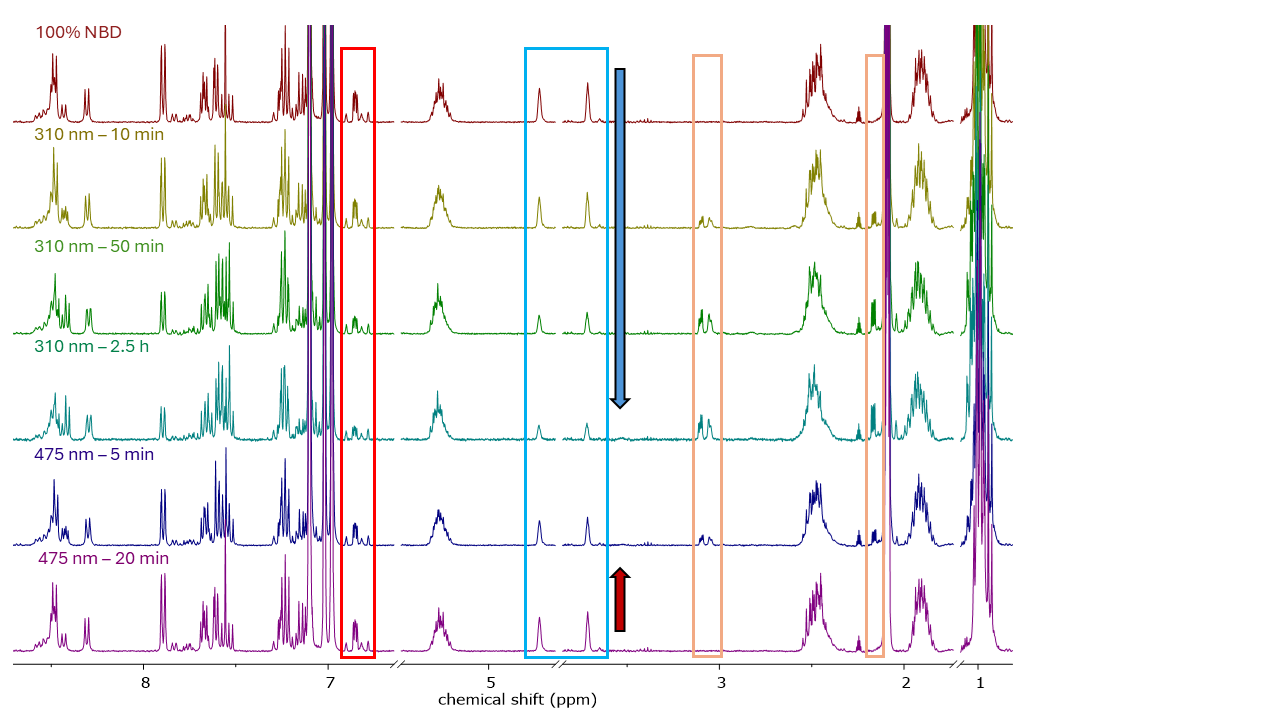


Figure SI 50: Interconversion of **14** in **Tol-d_8_**. Red=NBD olefinic proton signals; blue=NBD bridgehead proton signals; orange=QC signals.


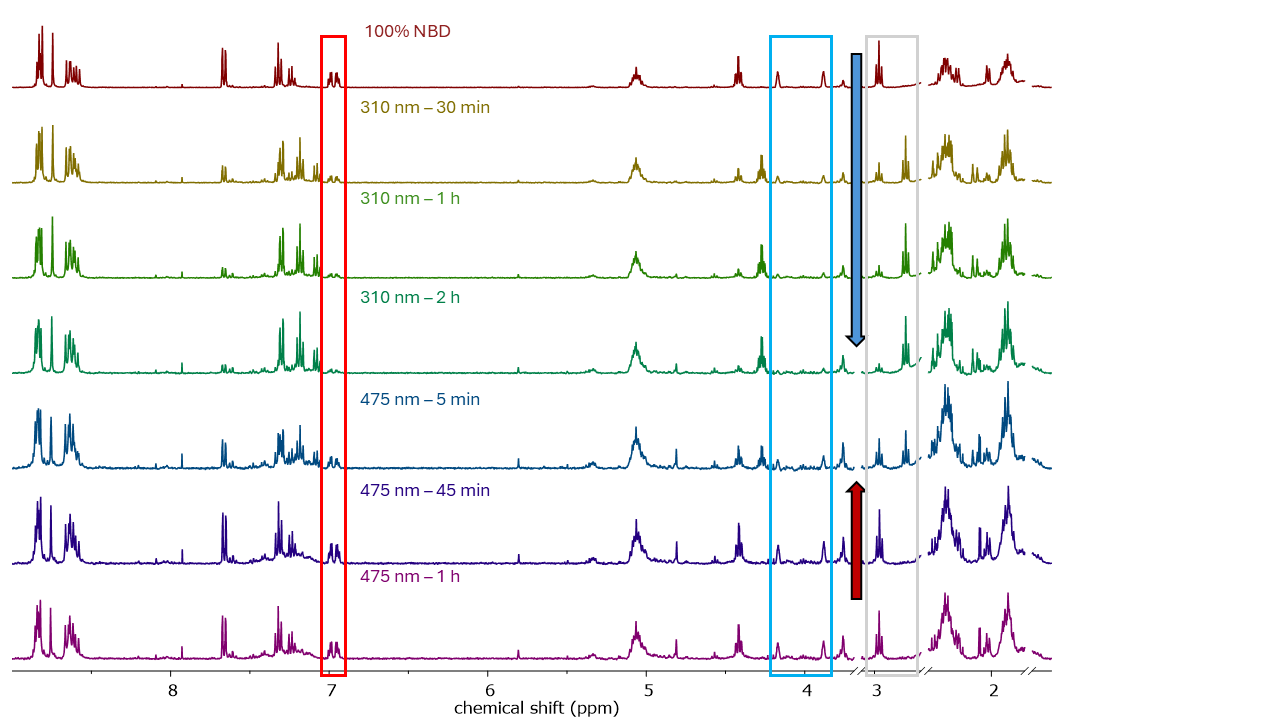


Figure SI 51: Interconversion of **15** in **THF-d_8_**. Red=NBD olefinic proton signals; blue=NBD bridgehead proton signals; grey=linker proton signals.


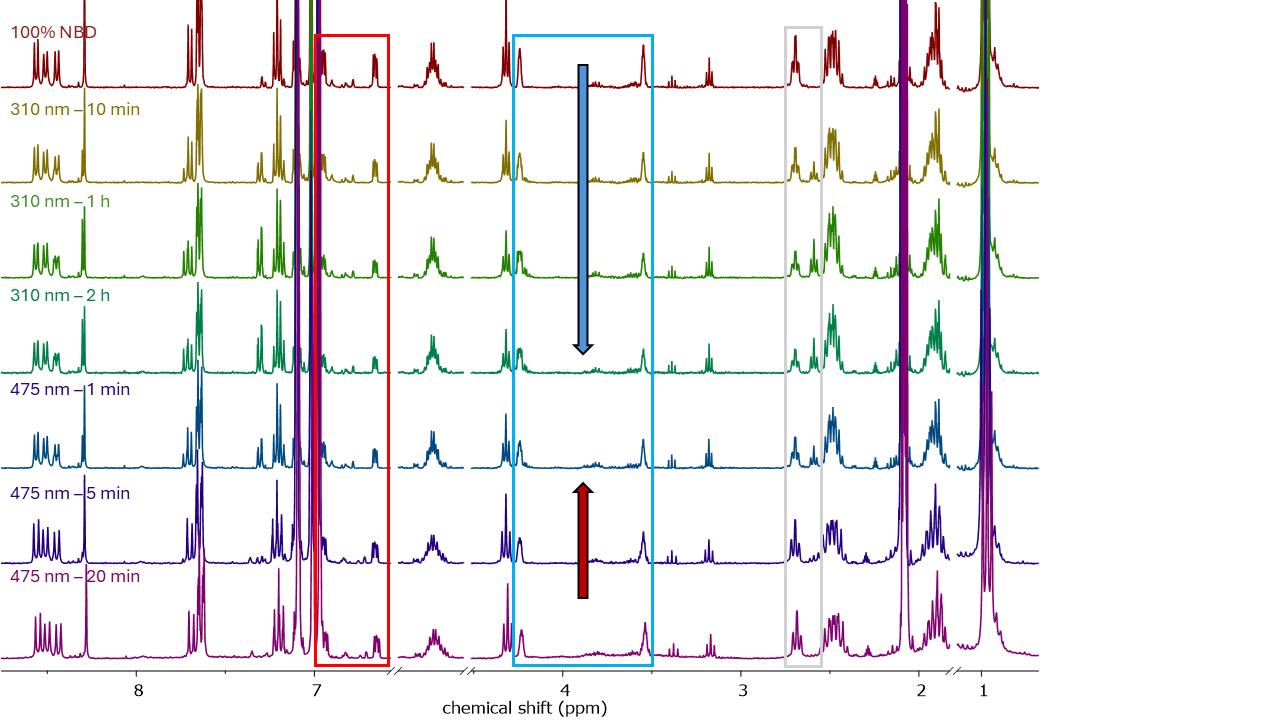


Figure SI 52: Interconversion of **15** in **Tol-d_8_**. Red=NBD olefinic proton signals; blue=NBD bridgehead proton signals; grey=linker proton signals; orange=QC signals.

Table SI 3: Time progression of the NMR‑monitored interconversion at the given irradiation wavelength in **THF-d_8_**. Maximum conversion to QC (red) and regeneration of NBD (blue):

| 12 to 16 (310 nm) | | 16 to 12 (475 nm) | | 13 to 17 (310 nm) | | 17 to 13 (475 nm) | |
| --- | --- | --- | --- | --- | --- | --- | --- |
| *t [min]* | *NBD [%]* | *t [min]* | *NBD [%]* | *t [min]* | *NBD [%]* | *t [min]* | *NBD [%]* |
| 0 | 100 | 10 | 56 | 0 | 100 | 10 | 40 |
| 30 | 63 | 30 | **77** | 30 | 63 | 45 | 78 |
| 120 | **19** |  |  | 80 | **13** | 105 | **92** |
| 14 to 18 (310 nm) | | **18 to 14 (475 nm)** | | **15 to 19 (310 nm)** | | **19 to 15 (475 nm)** | |
| *t [min]* | *NBD [%]* | *t [min]* | *NBD [%]* | *t [min]* | *NBD [%]* | *t [min]* | *NBD [%]* |
| 0 | 100 | 5 | 29 | 0 | 100 | 5 | 20 |
| 10 | 78 | 20 | **67** | 30 | 16 | 45 | **100*** |
| 50 | 40 |  |  | 60 | 8 |  |  |
| 210 | **15** |  |  | 120 | **1** |  |  |

*The initial NBD signal integrals were not regenerated, but the QC signals vanished completely. Decomposition is assumed.

Table SI 4: Time progression of the NMR‑monitored interconversion at the given irradiation wavelength in **Tol-d_8_**. Maximum conversion to QC (red) and regeneration of NBD (blue):

| 12 to 16 (310 nm) | | 16 to 12 (475 nm) | | 13 to 17 (310 nm) | | 17 to 13 (475 nm) | |
| --- | --- | --- | --- | --- | --- | --- | --- |
| *t [min]* | *NBD [%]* | *t [min]* | *NBD [%]* | *t [min]* | *NBD [%]* | *t [min]* | *NBD [%]* |
| 0 | 100 | 10 | 85 | 0 | 100 | 10 | 69 |
| 30 | 73 | 30 | **96** | 30 | 73 | 30 | **96** |
| 120 | **35** |  |  | 165 | **21** |  |  |
| 14 to 18 (310 nm) | | **18 to 14 (475 nm)** | | **15 to 19 (310 nm)** | | **19 to 15 (475 nm)** | |
| *t [min]* | *NBD [%]* | *t [min]* | *NBD [%]* | *t [min]* | *NBD [%]* | *t [min]* | *NBD [%]* |
| 0 | 100 | 5 | 74 | 0 | 100 | 1 | 70 |
| 10 | 77 | 20 | **100** | 10 | 73 | 5 | 85 |
| 50 | 48 |  |  | 60 | 55 | 20 | **94** |
| 150 | **40** |  |  | 120 | **41** |  |  |

Computational details

All calculations were performed with the Gaussian 16, Revision C.01 program package.^[3]^ Based on previous functional comparisons,^[4]^ the combination of the B3LYP hybrid functional^[5–9]^ and the triple-zeta 6-311++G(d,p)^[10,11]^ basis set was chosen for the presented calculations. Dispersion forces were taken into account using Grimme´s D3 correction with Becke-Johnson damping parameters.^[12,13]^ All geometries were optimized without any restrictions or solvent corrections and verified as true minima by vibrational frequency analysis and the absence of negative eigenvalues.

Conformer searches for all synthesized PDI-NBD hybrids were performed using the xtb 6.4.1 program^[14,15]^ and the Conformer Rotamer Ensemble Sampling Tool (CREST, Version 2.11.2)^[16–18]^ with the GFN2‑xTB method.^[19]^ Conformer search was carried out with the improved metadynamic sampling (iMTD) and genetic Z-matrix crossing^[16,17]^ with an energy threshold of 15 kcal/mol and a RMSD threshold of 1 Å. The obtained conformers with relative energies up to 2 kcal/mol above the lowest energy conformer were optimized using B3LYP/6-311++G(d,p). The lowest energy conformer determined by this method was used for the calculation of the respective QC isomer. The published structures of bay-substituted compounds and NDI-NBD were used as initial coordinates for reoptimization at the method chosen for this work.

Results were visualized using GaussView,^[20]^ Molden,^[21]^ PyMOL^[22]^ and Origin 2024. Frontier molecular orbitals are shown at an isovalue of 0.02. The cartesian coordinates of the calculated molecules are provided in a separate file.

Optimized geometries with selected bond lengths (in Å) and dihedrals. *Bay*‑substituted PDIs (PDI‑*bay*‑Est- and PDI‑*bay*‑Ac-) and NDI‑NBD were calculated and are depicted for comparison.


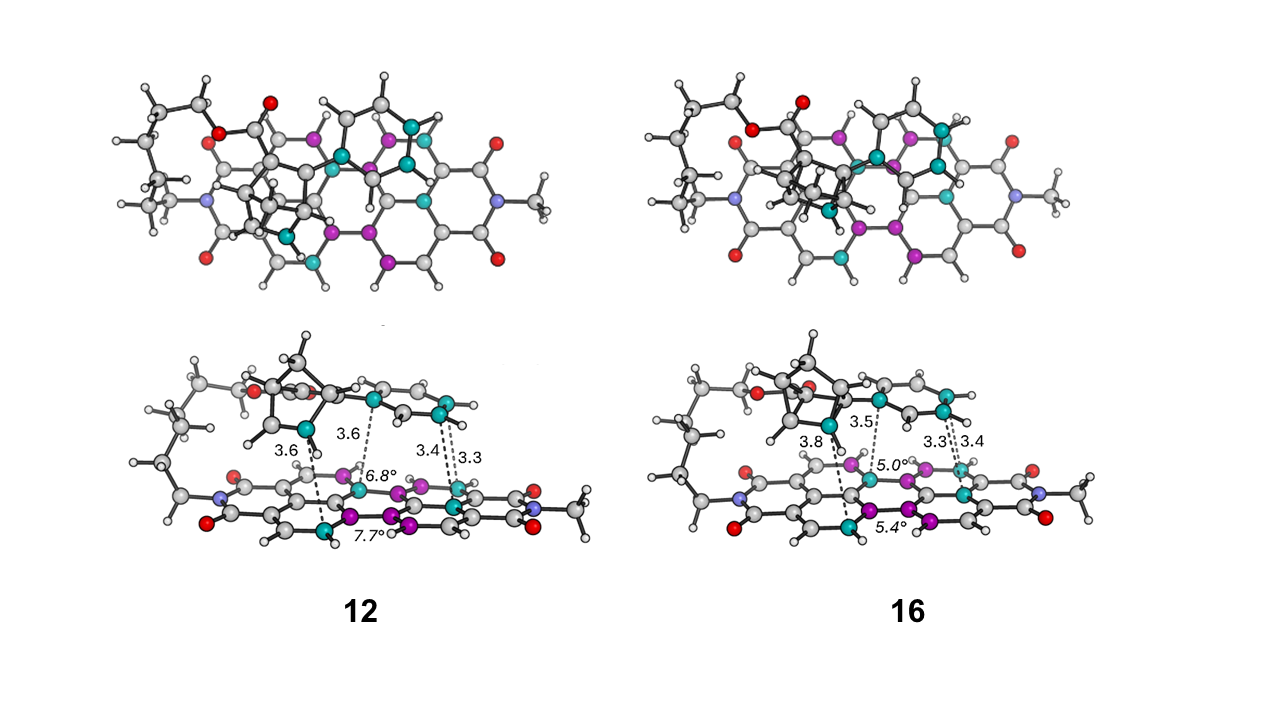


Figure SI 53: Optimized structures of **12** and **16** in top view (top) and side view (bottom).


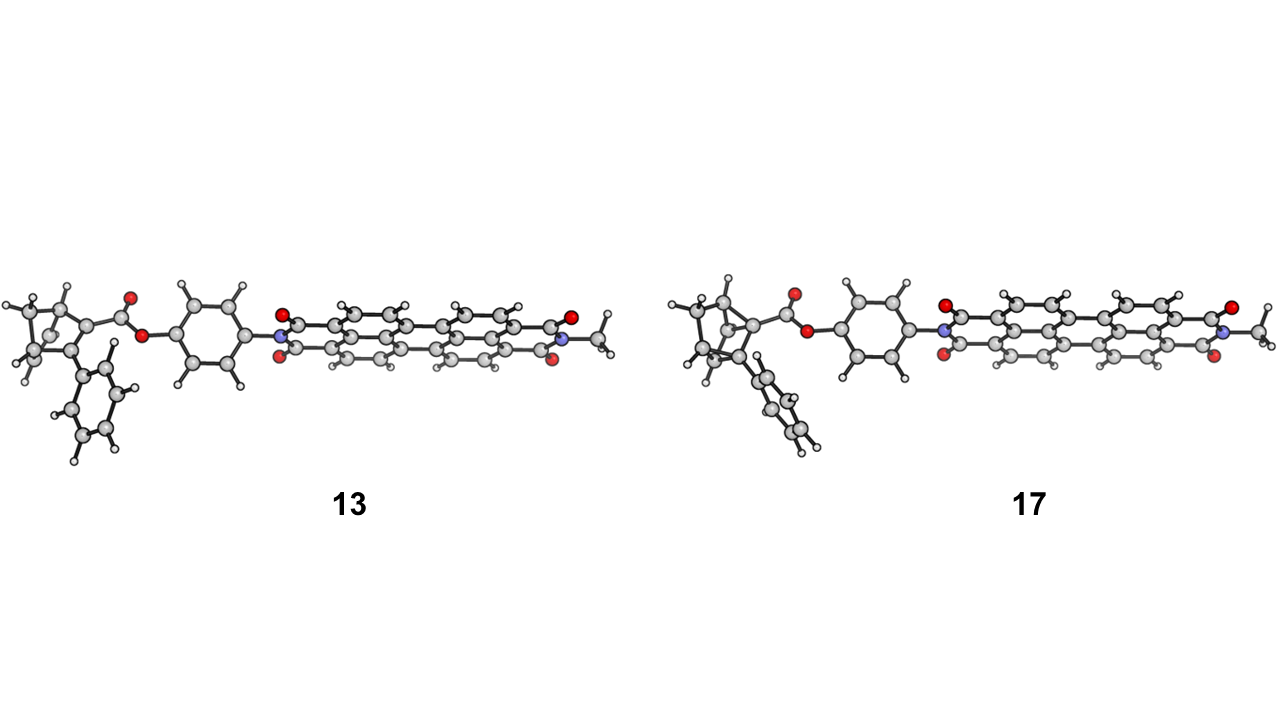


Figure SI 54: Optimized structures of **13** and **17**.


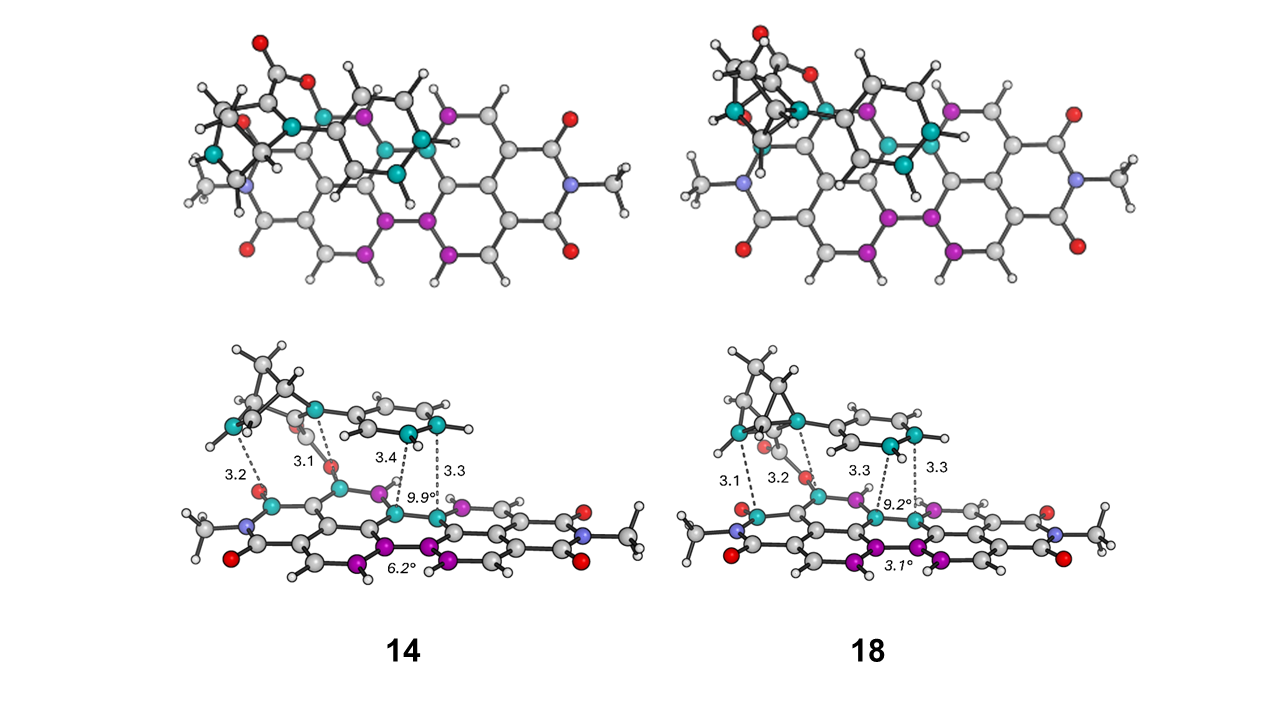


Figure SI 55: Optimized structures of **14** and **18** in top view (top) and side view (bottom).


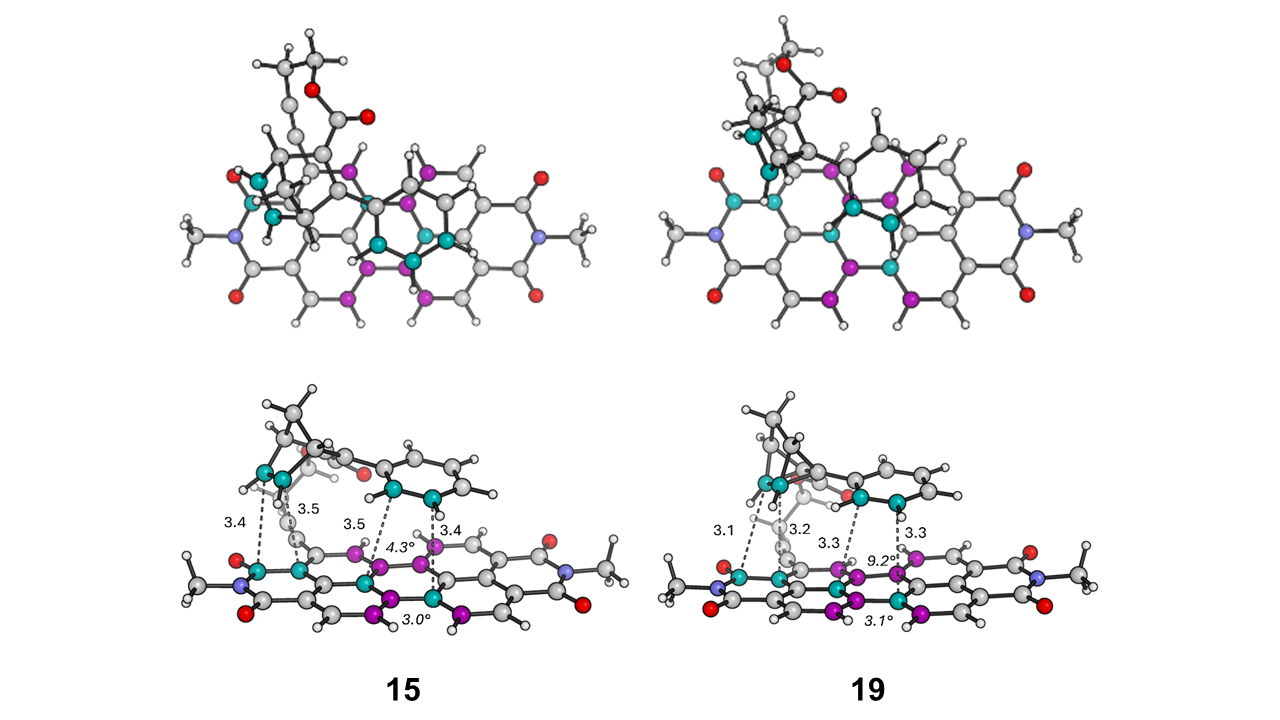


Figure SI 56: Optimized structures of **15** and **19** in top view (top) and side view (bottom).


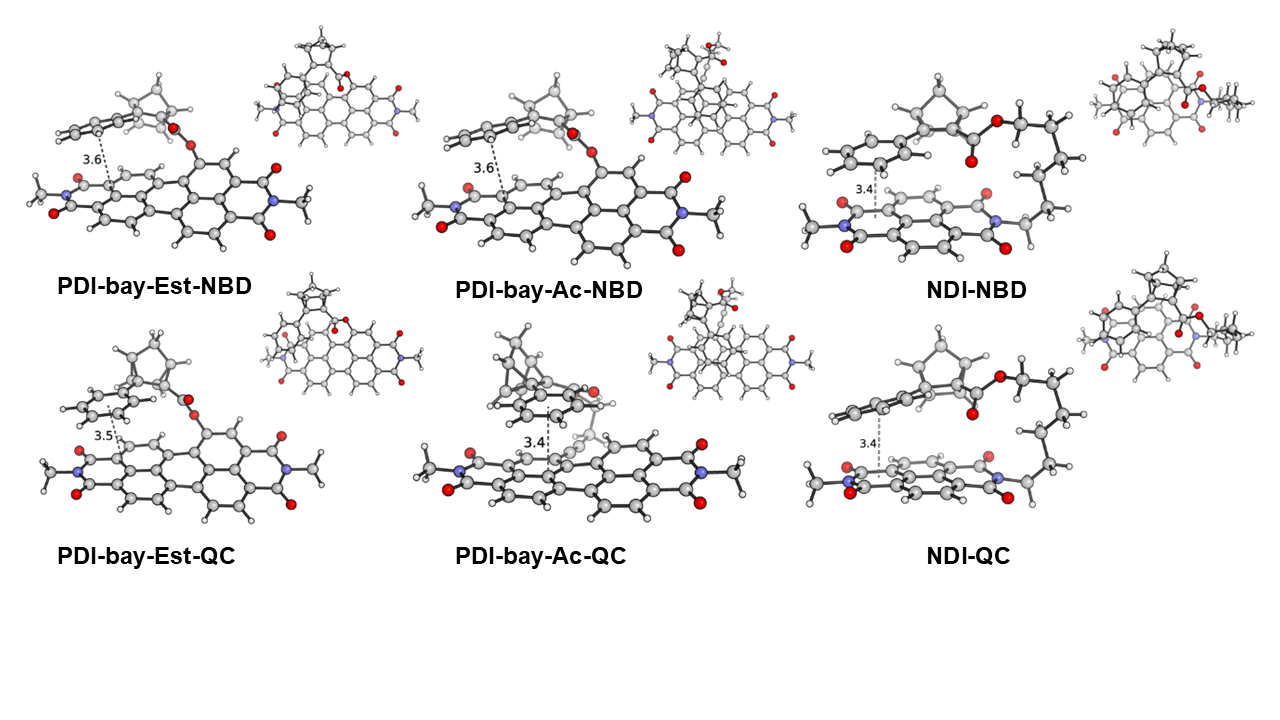

Figure SI 57: Optimized structures of previously reported bay-substituted PDI-bay-Est-NBD and PDI-bay-Ac-NBD as well as NDI-NBD for comparison.

Frontier Molecular Orbitals (FMOs)


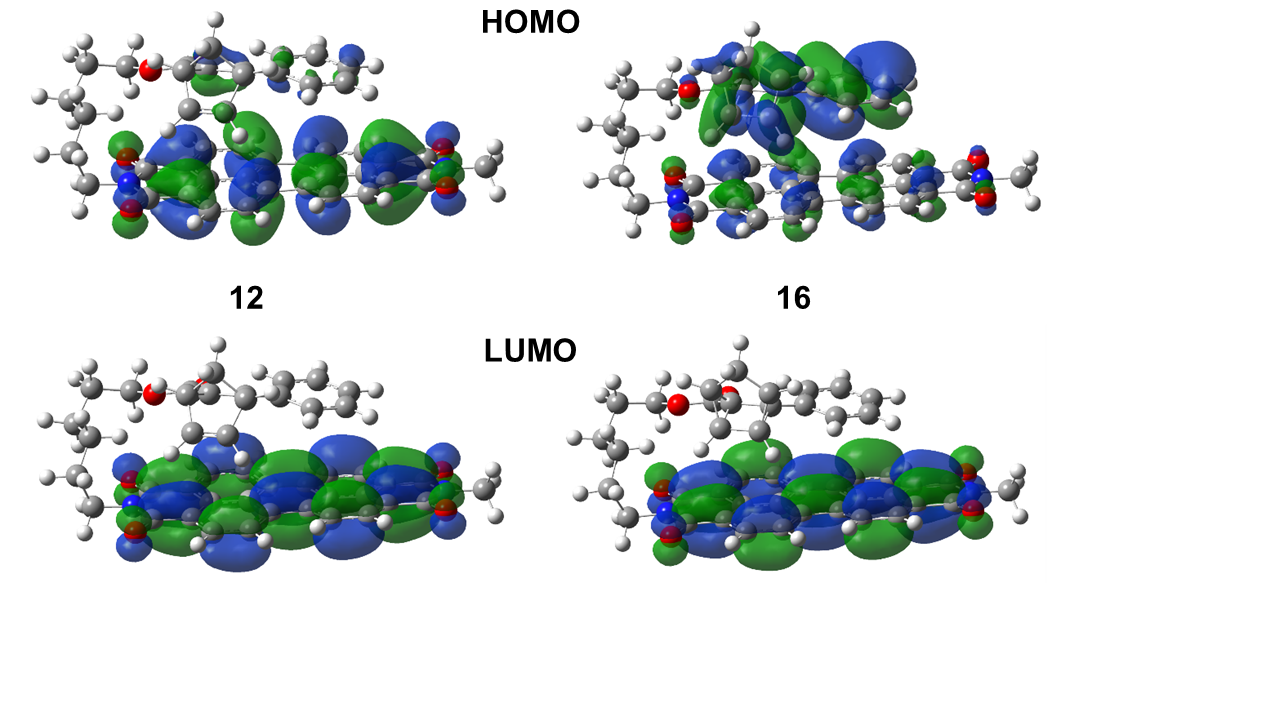


Figure SI 58: Visualization of the lowest energy FMOs of **12** and **16**.


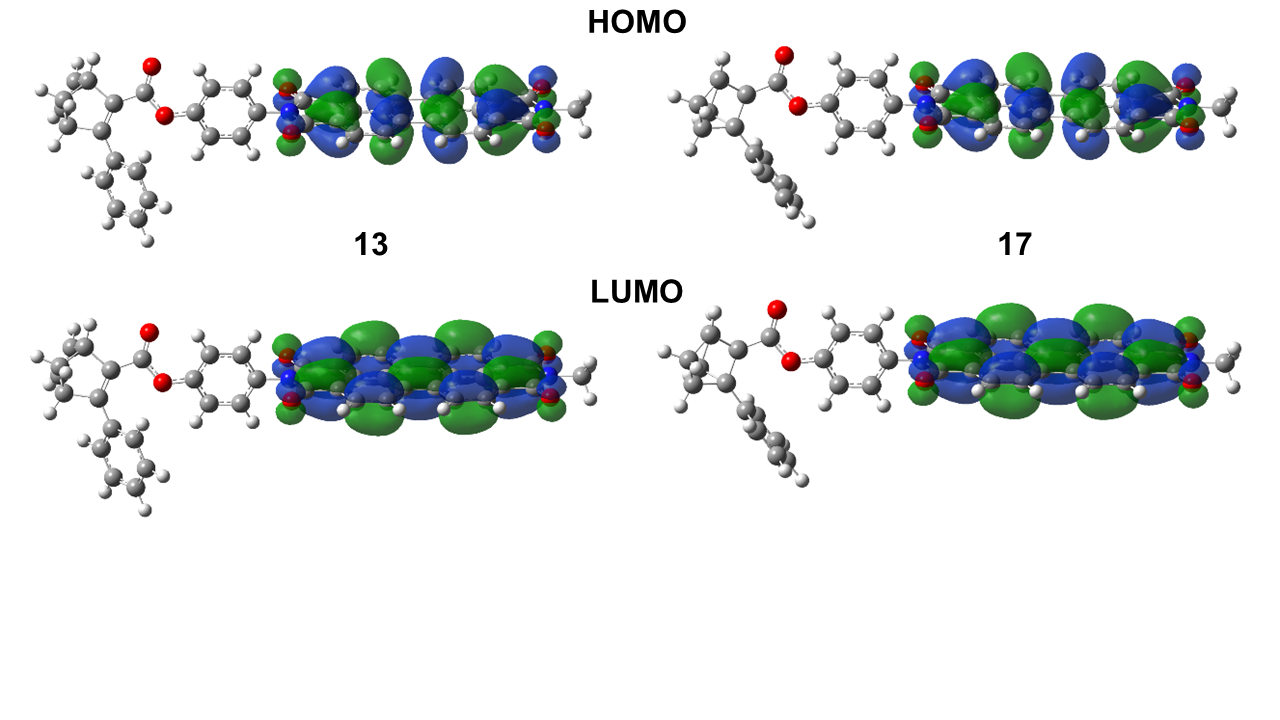


Figure SI 59: Visualization of the lowest energy FMOs of **13** and **17**.


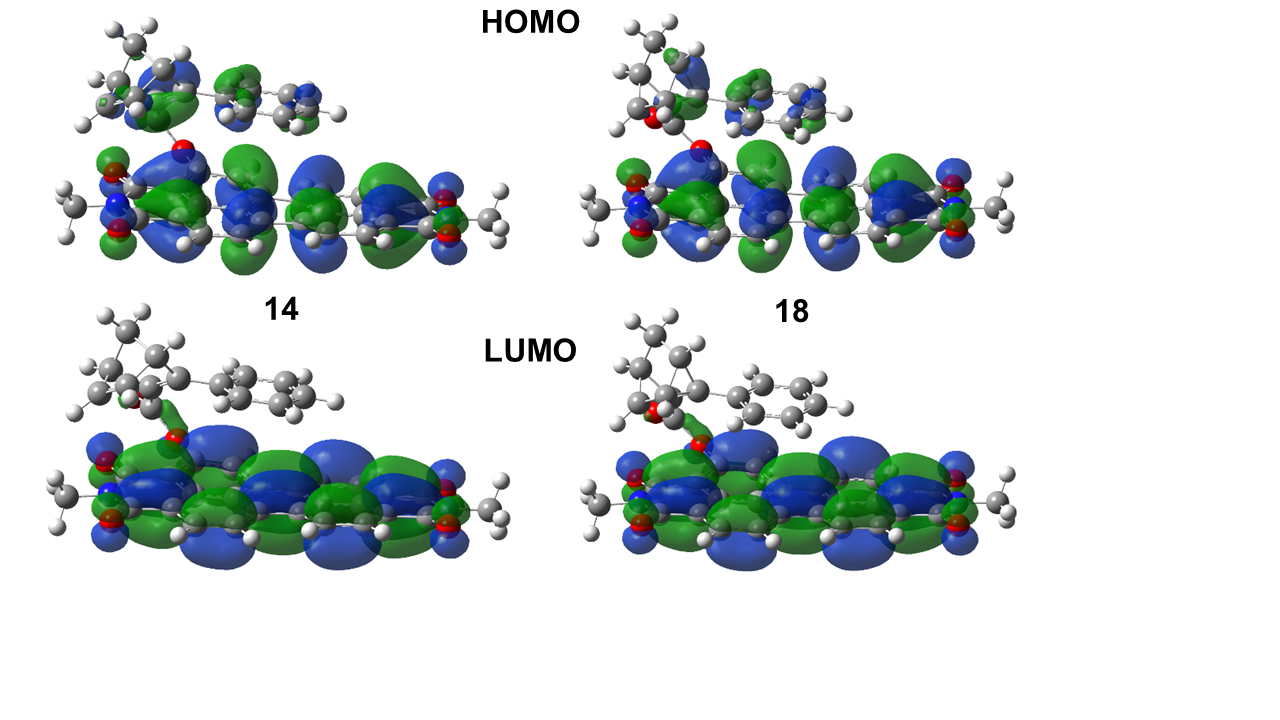


Figure SI 60: Visualization of the lowest energy FMOs of **14** and **18**.


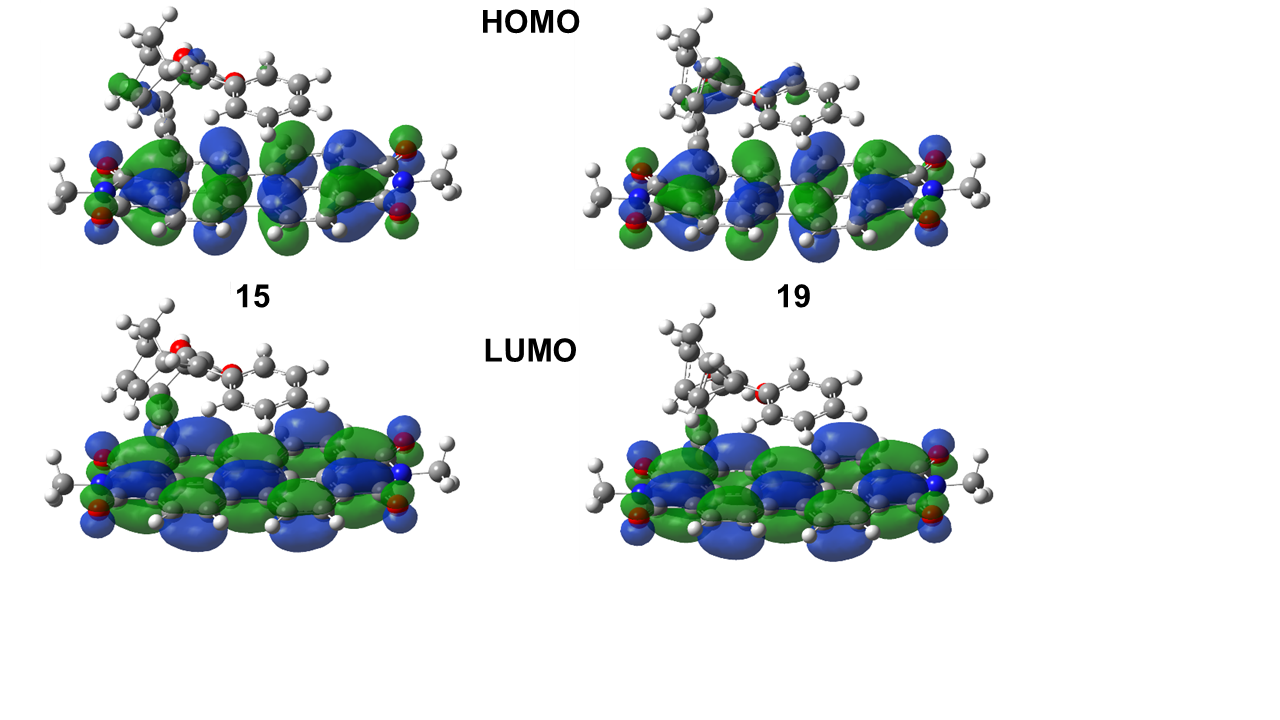


Figure SI 61: Visualization of the lowest energy FMOs of **15** and **19**.

Table SI 5: Energy storage capacity (ΔH) in kcal/mol and HOMO-LUMO gaps in eV (B3LYP/6-311++G(d,p) D3BJ).

|  | ΔH (QC - NBD) | HOMO-LUMO gap (NBD/QC) |
| --- | --- | --- |
| **12** | 26.9 | 2.5/ 2.4 |
| **13** | 24.6 | 2.5/ 2.5 |
| **14** | 28.7 | 2.5/ 2.5 |
| **15** | 26.3 | 2.5/ 2.5 |
| **PDI-*bay*-Ac-** | 27.2 | 2.4/ 2.4 |
| **PDI-*bay*-Est-** | 28.2 | 2.5/ 2.5 |

**References**

[1] E. Marinho, P. R. Figueiredo, R. Araújo, M. F. Proença, "A simple protocol for the synthesis of perylene bisimides from perylene tetracarboxylic dianhydride", *RSC Adv.* **2024**, *14*, 11141–11150.

[2] P. Lorenz, T. Luchs, A. Hirsch, "Molecular Solar Thermal Batteries through Combination of Magnetic Nanoparticle Catalysts and Tailored Norbornadiene Photoswitches", *Chem. Eur. J.* **2021**, *27*, 4993–5002.

[3] M. J. Frisch, G. W. Trucks, H. B. Schlegel, G. E. Scuseria, M. A. Robb, J. R. Cheeseman, G. Scalmani, V. Barone, G. A. Petersson, H. Nakatsuji, X. Li, M. Caricato, A. V. Marenich, J. Bloino, B. G. Janesko, R. Gomperts, B. Mennucci, H. P. Hratchian, J. V. Ortiz, A. F. Izmaylov, J. L. Sonnenberg, Williams, F. Ding, F. Lipparini, F. Egidi, J. Goings, B. Peng, A. Petrone, T. Henderson, D. Ranasinghe, V. G. Zakrzewski, J. Gao, N. Rega, G. Zheng, W. Liang, M. Hada, M. Ehara, K. Toyota, R. Fukuda, J. Hasegawa, M. Ishida, T. Nakajima, Y. Honda, O. Kitao, H. Nakai, T. Vreven, K. Throssell, J. A. Montgomery Jr., J. E. Peralta, F. Ogliaro, M. J. Bearpark, J. J. Heyd, E. N. Brothers, K. N. Kudin, V. N. Staroverov, T. A. Keith, R. Kobayashi, J. Normand, K. Raghavachari, A. P. Rendell, J. C. Burant, S. S. Iyengar, J. Tomasi, M. Cossi, J. M. Millam, M. Klene, C. Adamo, R. Cammi, J. W. Ochterski, R. L. Martin, K. Morokuma, O. Farkas, J. B. Foresman, D. J. Fox, *Gaussian 16 Rev. C.01*, Wallingford, CT, **2015**.

[4] M. J. Kuisma, A. M. Lundin, K. Moth-Poulsen, P. Hyldgaard, P. Erhart, "Comparative Ab-Initio Study of Substituted Norbornadiene-Quadricyclane Compounds for Solar Thermal Storage", *J. Phys. Chem. C* **2016**, *120*, 3635–3645.

[5] A. D. Becke, "A new mixing of Hartree-Fock and local density-functional theories", *J. Chem. Phys.* **1993**, *98*, 1372–1377.

[6] C. Lee, W. Yang, R. G. Parr, "Development of the Colle-Salvetti correlation-energy formula into a functional of the electron density", *Phys. Rev. B* **1988**, *37*, 785–789.

[7] B. Miehlich, A. Savin, H. Stoll, H. Preuss, "Results obtained with the correlation energy density functionals of Becke and Lee, Yang and Parr", *Chem. Phys. Lett.* **1989**, *157*, 200–206.

[8] P. J. Stephens, F. J. Devlin, C. F. Chabalowski, M. J. Frisch, "Ab Initio calculation of vibrational absorption and circular dichroism spectra using density functional force fields", *J. Phys. Chem.* **1994**, *98*, 11623–11627.

[9] S. H. Vosko, L. Wilk, M. Nusair, "Accurate spin-dependent electron liquid correlation energies for local spin density calculations: a critical analysis", *Can. J. Phys.* **1980**, *58*, 1200–1211.

[10] R. Krishnan, J. S. Binkley, R. Seeger, J. A. Pople, "Self‐consistent molecular orbital methods. XX. A basis set for correlated wave functions", *J. Chem. Phys.* **1980**, *72*, 650–654.

[11] A. D. McLean, G. S. Chandler, "Contracted Gaussian basis sets for molecular calculations. I. Second row atoms, Z=11-18", *J. Chem. Phys.* **1980**, *72*, 5639–5648.

[12] S. Grimme, J. Antony, S. Ehrlich, H. Krieg, "A consistent and accurate ab initio parametrization of density functional dispersion correction (DFT-D) for the 94 elements H-Pu", *J. Chem. Phys.* **2010**, *132*, 154104.

[13] S. Grimme, S. Ehrlich, L. Goerigk, "Effect of the damping function in dispersion corrected density functional theory", *J. Comput. Chem.* **2011**, *32*, 1456–1465.

[14] S. Grimme, , “GitHub Semiempirical Extended Tight-Binding Program Package,” can be found under https://github.com/grimme-lab/xtb, **2020**.

[15] C. Bannwarth, E. Caldeweyher, S. Ehlert, A. Hansen, P. Pracht, J. Seibert, S. Spicher, S. Grimme, "Extended tight-binding quantum chemistry methods", *Wiley Interdiscip. Rev. Comput. Mol. Sci.* **2021**, *11*, e1493.

[16] P. Pracht, F. Bohle, S. Grimme, "Automated exploration of the low-energy chemical space with fast quantum chemical methods", *Phys. Chem. Chem. Phys.* **2020**, *22*, 7169–7192.

[17] S. Grimme, "Exploration of Chemical Compound, Conformer, and Reaction Space with Meta-Dynamics Simulations Based on Tight-Binding Quantum Chemical Calculations", *J. Chem. Theory Comput.* **2019**, *15*, 2847–2862.

[18] C. Bannwarth, S. Ehlert, S. Grimme, "GFN2-xTB - An Accurate and Broadly Parametrized Self-Consistent Tight-Binding Quantum Chemical Method with Multipole Electrostatics and Density-Dependent Dispersion Contributions", *J. Chem. Theory Comput.* **2019**, *15*, 1652–1671.

[19] R. Dennington, T. Keith, J. Millam, GaussView, Version 6.0, *Semichem Inc.*, *Shawnee Missions KS.*

[20] G. Schaftenaar, J. H. Noordik, "Molden: A pre- and post-processing program for molecular and electronic structures", *J. Comput. Aided. Mol. Des.* **2000**, *14*, 123–134.

[21] Schrodinger, LLC, The PyMOL Molecular Graphics System, Version 2.4.1., **2015**.
